# Integration ATAC‐Seq and RNA‐Seq Analysis of Mammary Placodes in Erhualian and Bamaxiang Pigs Identified Candidate Genes Influencing Pig Teat Number Variation

**DOI:** 10.1111/eva.70129

**Published:** 2025-06-28

**Authors:** Chenxi Liu, Ruihua Huang, Nengjing Jiang, Wuduo Zhou, Qian Liu, Taoran Du, Qian Zhang, Jinfeng Ma, Qingbo Zhao, Pinghua Li

**Affiliations:** ^1^ Institute of Swine Science (Key Laboratory of Pig Genetic Resources Evaluation and Utilization, Ministry of Agriculture and Rural Affairs (Nanjing)) Nanjing Agricultural University Nanjing People's Republic of China; ^2^ College of Animal Science and Technology Hunan Agricultural University Changsha People's Republic of China; ^3^ Huaian Academy, Nanjing Agricultural University Huaian People's Republic of China

**Keywords:** ATAC‐seq, Bamaxiang pigs, Erhualian pigs, mammary placodes, RNA‐seq

## Abstract

Teat number is an important economic trait in pigs, affecting piglet health and survival. While numerous GWAS have identified candidate genes for teat number in Duroc, Landrace, and Large White pigs, the causal genes remain unclear, largely due to a lack of transcriptional and epigenetic studies on mammary placodes in 26‐day‐old pig embryos, a critical stage for teat formation. Erhualian and Bamaxiang pigs, derived from Chinese wild boars, serve as ideal models for studying genetic variation in teat number, with Erhualian averaging nearly 20 teats and Bamaxiang around 10. This study collected mammary placodes from these breeds at embryonic day 26 and performed ATAC‐seq and RNA‐seq to explore chromatin accessibility and gene expression. Results indicate widespread chromatin accessibility across mammary placodes. Of the 30,806 open chromatin regions (OCRs) identified, only 30 showed breed‐specific differences, suggesting conserved accessibility patterns across breeds. OCRs are enriched in intergenic and promoter regions, and significantly overlap with QTL intervals for teat number. RNA‐seq revealed 4432 differentially expressed genes between the two breeds, including *WTN10B* and *WNT6*, indicating breed‐specific gene expression patterns. Combining ATAC‐seq and RNA‐seq results identified three protein‐coding genes (*ENSSSCG00000031037*, *ENSSSCG00000032042*, and *ENSSSCG00000039180*) near 48.80 Mb on SSC14 that are associated with teat number according to pheWAS and GWAS data. FISH analysis confirmed that *ENSSSCG00000031037* is specifically expressed in epithelial cells of mammary placodes, and this region is under stronger selection in Erhualian pigs, suggesting its role in the breed's higher teat number. In conclusion, this study integrates ATAC‐seq and RNA‐seq to construct a chromatin accessibility and gene expression map of pig mammary placodes. It identifies *ENSSSCG00000031037*, *ENSSSCG00000032042*, and *ENSSSCG00000039180* as key candidate genes driving teat number differences, providing insights for understanding QTL intervals and identifying causal genes linked to teat number in pigs.

## 1 Introduction

Teat number is a crucial factor influencing pig reproductive performance. Higher teat numbers are generally associated with enhanced maternal nursing ability and higher piglet survival rates. Additionally, ensuring piglets receive adequate nutrition immediately after birth is vital for their early growth and development, significantly enhancing feed efficiency and final market weight (Chu et al. 2022; C et al. 2013; KilBride et al. 2014). However, with the application of genomic selection technologies, the trait of litter size in different pig breeds has been continually enhanced, often resulting in a teat count lower than the number of offspring (Kobek‐Kjeldager et al. 2021). This discrepancy significantly impacts piglet health and growth, underscoring the urgent need to identify key genes and functional loci influencing variations in pig teat numbers for breeding enhancement.

Although numerous studies have identified candidate genes affecting teat number in Duroc, Landrace, and Large White pigs through genome‐wide association analyses (GWAS) (Bovo et al. 2021; Zeng et al. 2024), the causal genes have yet to be identified. This is due to the lack of research on transcriptional expression and epigenetics in key tissues involved in teat formation, such as mammary placodes at embryonic day 26. Furthermore, some studies have identified genes affecting the development of the pig mammary gland postbirth (Martínez‐Giner et al. 2011; Moss et al. 2020). However, for various mammals, the critical period determining teat number is early embryogenesis, specifically during the formation and development of the mammary placodes. Each mammary placode, once properly developed, forms a corresponding teat (Chu et al. 2004; Mailleux et al. 2002; Sakai et al. 2019; Spina and Cowin 2021). Specifically for pigs, the formation of early embryonic mammary glands occurs through the following four stages: (1) Formation of the mammary line: Around day 23 of pig embryogenesis, the mammary line is formed by columnar and multilayered ectodermal cells along the sides of the abdomen; (2) Formation of mammary placodes: Within 24–36 h after the formation of the mammary line, around day 26 of embryogenesis, the mammary line progressively specializes into clearly visible mammary placodes, a critical developmental stage determining the number of pig teats; (3) Formation of gland buds: The mammary substratum proliferates and indents into the underlying mesenchyme, forming bud‐like structures known as gland buds; (4) Formation of primary mammary ductal branching, constituting the primary mammary gland (Zhou et al. 2019). Therefore, to determine the key genes and functional loci responsible for differences in pig teat counts at the molecular level, it is necessary to integrate the gene expression maps of mammary placodes.

Currently, research on mammary placodes primarily focuses on mice and rabbits (Chu et al. 2004; González‐Mariscal et al. 2013; Mailleux et al. 2002; Propper et al. 2013; Sakai et al. 2019; Spina and Cowin 2021; Veltmaat et al. 2003, 2004, 2006), with several important pathways identified that influence the formation and development of mammary placodes. The most significant of these is the Wingless/Integrated (WNT) signaling pathway. In mouse studies, *Wnt10b* expression begins at embryo day (E)10.25, with distinct *Wnt10b* expression lines also appearing in the axillary and inguinal (groin) regions, where the first and fifth mouse mammary placodes will form. Within 1 day of embryonic development, the intensity of *Wnt10b* expression increases. Subsequently, the lines split into individual points, and by E12, expression is confined to the forming mammary placodes. The expression of *Wnt6* and *Wnt10a* follows a similar pattern (Veltmaat et al. 2004). Along with the expression of the WNT family genes, the formation of mammary placodes is driven by the canonical WNT‐β‐catenin signaling pathway (Chu et al. 2004). Additionally, fibroblast growth factor (FGF) signaling from the rib plate is crucial for initiating mammary cell fate, with members of the FGF gene family such as *FGF10* being essential for inducing mammary cell fate (Veltmaat et al. 2006). Moreover, the epidermal growth factor receptor (EGFR) family signaling pathway can influence the number and location of mammary placodes (Howard et al. 2005). Activation of Hedgehog signaling results in aberrant development of the mouse ectodermal mammary (Gritli‐Linde et al. 2007). Notch signaling regulates interactions between epithelial cells and mesenchymal cells, playing a crucial role in the formation and differentiation of mammary ducts (Buono et al. 2006). This signaling pathways provide critical references for a systemic understanding of the formation of mammalian mammary glands.

In elucidating the complex quantitative genetic mechanisms of traits such as pig teat number, RNA‐seq provides robust tissue expression profiles. Additionally, epigenetic‐based high‐throughput sequencing technologies, such as assay for transposase‐accessible chromatin using sequencing (ATAC‐seq) (Luo et al. 2022), cleavage under targets and tagmentation (CUT&TAG) (Kaya‐Okur et al. 2019), and DNA methylation sequencing (Corbett et al. 2021), effectively link the genome with transcriptomics. These methods not only facilitate the screening of key genes but also elucidate the epigenetic regulatory mechanisms behind gene expression differences. Zhang, Liu, et al. (2023) integrated RNA‐seq and ATAC‐seq data from the endometrial tissues of Meishan and Duroc pigs to identify candidate genes affecting swine reproductive performance, such as *ANXA4*. Quan et al. integrated methylation, chromatin accessibility, and gene expression data to demonstrate that allele‐specific expression can largely be explained by allele‐specific methylation and chromatin accessibility (Quan et al. 2024).

Currently, there is a lack of exploration into the chromatin accessibility patterns of pig mammary placodes, and research related to the transcriptome of mammary placodes is limited. Erhualian pigs and Bamaxiang pigs are two representative Chinese indigenous pig breeds, both originating from Asian wild boars. Under long‐term artificial selection pressure, Erhualian pigs have developed traits such as high litter size and increased teat number, while Bamaxiang pigs, subjected to different selection pressures (China National Committee on Animal Genetic Resources 2011), exhibit a smaller body size, lower litter size, and reduced teat number. In terms of teat number, Erhualian pigs have an average of 20 teats, ranking among the highest in Chinese indigenous pig breeds and globally. In contrast, Bamaxiang pigs have an average of 10–12 teats, placing them at the lower end among Chinese indigenous pig breeds. Therefore, using Erhualian and Bamaxiang pigs to study the gene architecture underpinning teat number is advantageous for several reasons: first, as Chinese indigenous breeds, they share a close genetic origin. Second, their significant phenotypic divergence in teat number makes them an ideal model for studying teat number variation. This approach allows for the accurate identification of genetic mechanisms underlying teat number variation while minimizing the influence of genetic background differences. Therefore, in this study, we selected Erhualian and Bamaxiang pigs as research subjects and collected mammary placodes from these two breeds for RNA‐seq and ATAC‐seq analyses. By initially exploring the gene expression patterns and chromatin accessibility spectra of pig mammary placodes, and through interbreed difference analysis combined with the aforementioned omics analyses, this study aims to identify key candidate genes responsible for the differences in teat number between breeds. These findings will provide insights for the breeding of different pig breeds for enhanced teat numbers.

## 2 Materials and Methods

### 2.1 Animal Population

**FIGURE 1:**
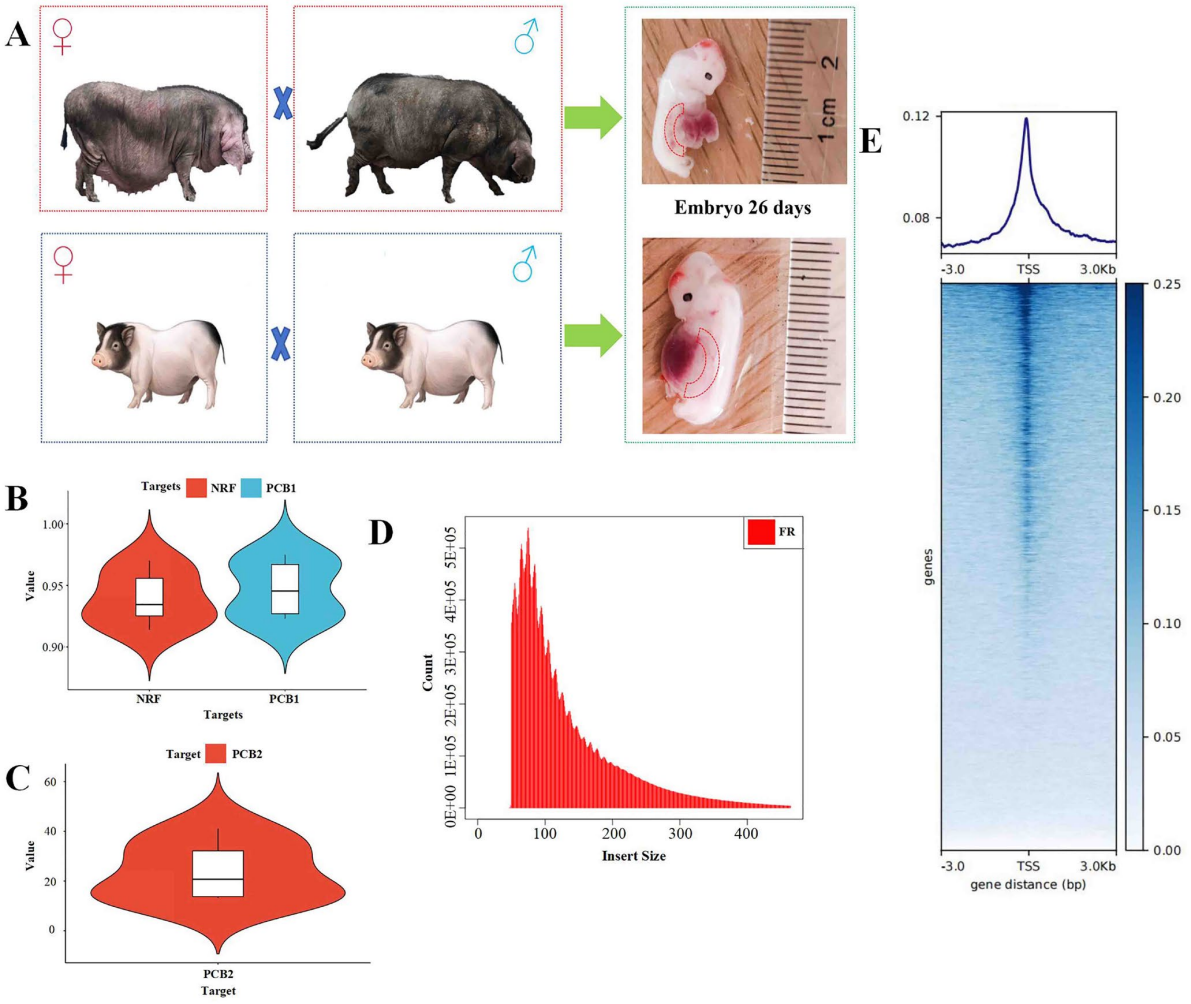
Quality statistics of ATAC‐seq in mammary placodes. (A) Sampling sites of mammary placodes at 26 days of embryonic development in Erhualian pigs (the above image) and Bamaxiang pigs (the below image). The red box indicates the anatomical location of the mammary placodes between the upper and lower limb buds. (B, C) ATAC‐seq sequencing quality statistics, where NRF represents the nonredundant fraction, calculated as NRF = number of uniquely aligned reads/total number of reads. PCB1 represents Phantom Peak Coefficient 1, calculated as PCB1 = number of uniquely aligned reads/number of reads aligned multiple times. PCB2 represents Phantom Peak Coefficient 2, calculated as PBC2 = number of singly aligned reads/total number of reads. (D) Statistics on the fragment size distribution of ATAC‐seq libraries. The Insert Size represents the length of sequencing reads. FR (Forward‐Reverse orientation) indicates the normal paired‐end sequencing orientation. (E) ATAC‐seq signal enrichment surrounding the TSS. The *y*‐axis represents the degree of enrichment of all genes at the TSS (Transcription Start Site), with enrichment gradually decreasing from top to bottom. The *x*‐axis represents the distance from the TSS, where negative values indicate upstream regions and positive values indicate downstream regions.

We conducted breeding experiments in Erhualian pigs, with all individuals sourced from a Erhualian pig farm in Changshu, Jiangsu Province. On approximately day 26 of embryogenesis, the pig mammary line progressively differentiates into distinct mammary placodes (Zhou et al. 2019). Based on this specific developmental stage in pig embryos, with the day of natural mating (between boar and sow) designated as postconception day 0, the sow was slaughtered at 26 days postconception, and a total of 20 embryos were collected. Similarly, breeding experiments were also conducted in Bamaxiang pigs, where all individuals were obtained from a Bamaxiang pig farm in Yangzhou, Jiangsu Province (Figure 1A). Bamaxiang sow was also slaughtered at 26 days after conception, resulting in a total of six embryos being collected. Based on the anatomical positions of the mammary placodes in mice and rabbits (Chu et al. 2004; Mailleux et al. 2002; Propper et al. 2013; Spina and Cowin 2021; Veltmaat et al. 2003, 2004, 2006), tissues from the ectoderm located between the upper and lower limb buds and the underlying mesenchyme were harvested as mammary placodes for subsequent experiments (Figure 1A).

### 2.2 ATAC‐Seq for Pig Mammary Placodes

For the mammary placodes collected from the Erhualian and Bamaxiang pig embryos at 26 days, three embryos from Erhualian pigs (EHL1, EHL2, EHL3) and three from Bamaxiang pigs (BMX1, BMX2, BMX3) were randomly selected for ATAC‐seq analysis. To determine the sex of the embryos, PCR amplification was performed targeting the pig *SRY* gene. Embryos amplifying the specific *SRY* gene fragment were identified as male, while those without amplification were identified as female. The primers used for the *SRY* gene were as follows: forward primer: CCGACGGACAATCATAGC; reverse primer: GGTGGATGTTACCCTACTGT. ATAC‐seq libraries were prepared from six samples where nuclei were isolated, subjected to TN5 transposase for tagmentation at 37°C for 30 min, followed by adapter ligation. After PCR amplification and AMpure bead purification, the quality of the libraries was assessed using Qubit. Index‐coded samples underwent cluster generation with the HiSeq PE Cluster Kit v4‐cBot‐HS (Illumina) and were sequenced on an Illumina platform to produce 150 bp paired‐end reads (Zhu et al. 2023). Raw sequencing images were converted into sequences recognized by bcl2fastq (v2.20) and saved in FASTQ format. Sequence quality refinement was performed using Trimmomatic (v0.36), aligned to the *Sus scrofa* 11.1 reference genome using Bowtie2 (v2.3.5) with the options “‐I 10 ‐X 1000 ‐‐dovetail ‐‐no‐unal ‐‐very‐sensitive‐local ‐‐no‐mixed ‐‐no‐discordant”, and open chromatin regions (OCRs) were mapped across the genome using MACS2 (v2.1.1) for peak detection with the options “‐f BAMPE ‐B ‐‐SPMR ‐‐keep‐dup 1 ‐p 0.001 ‐‐nomodel ‐‐shift ‐100 ‐‐extsize 200”.

The DiffBind R package was used to merge the peaks from a total of six samples from Erhualian and Bamaxiang pigs and to analyze differential peaks, or differential OCRs, between the breeds. Differential OCRs were identified with criteria of multiple testing using the false discovery rate (FDR) < 0.05. The dba.plotHeatmap function from DiffBind R was employed to generate a heatmap for individual clustering based on the peaks.

For the identified peaks, bedtools (v2.30.0) was used to intersect these with Quantitative Trait Locus (QTL) intervals from the pig QTL database to count overlaps. Fisher's exact test was applied to determine whether the peak regions were significantly enriched in teat number‐related QTL regions compared to expected values.

Transcription factor motif position frequency matrix data were downloaded from the JASPAR database (https://jaspar.genereg.net/downloads/) (Castro‐Mondragon et al. 2022). Subsequently, the AME (Analysis of Motif Enrichment) suite from MEME (v5.5.7) was used to conduct transcription factor motif enrichment analysis on the OCRs.

### 2.3 RNA‐Seq for Pig Mammary Placodes

For the mammary placodes collected from the Erhualian and Bamaxiang pig embryos at 26 days, the remaining 17 Erhualian pig embryos after ATAC‐seq were used, among which twelve randomly selected embryos were subjected to RNA‐seq. Additionally, the three remaining Bamaxiang pig embryos after ATAC‐seq were used for RNA‐seq analysis. The sex of the embryos was determined using PCR amplification of the *SRY* gene, with primers described in section 2.2 ATAC‐seq for pig mammary placodes. Total RNA was extracted from mammary placodes using the standard Trizol method. RNA library construction, sequencing, and alignment for each sample followed the procedures described in Liu et al. (Liu et al. 2023). Differentially expressed genes (DEGs) were identified with criteria of FDR < 0.05 and |log2 (fold change)| ≥ 1. For differentially expressed genes, the Kyoto Encyclopedia of Genes and Genomes (KEGG) functional enrichment analysis was performed using the KOBAS database (http://kobas.cbi.pku.edu.cn/kobas3) (Bu et al. 2021). The analysis was based on hypergeometric distribution statistics to determine whether the observed number of genes enriched in a specific pathway was significantly higher than expected. *p*‐values were calculated and subsequently corrected using the Benjamini‐Hochberg method for FDR multiple testing. An FDR < 0.05 was considered as the threshold for significantly enriched KEGG pathway.

### 2.4 Protein–Protein Interactions Analysis for Candidate Transcription Factors and Differentially Expressed Genes

We used the STRING database to predict protein–protein interactions (PPI) for the transcription factors *OLIG2* and *NEUROD2* enriched from differential peaks, as well as for differentially expressed genes (Franceschini et al. 2013). A combined score of ≥ 0.4 was selected for constructing the PPI network. The Cytoscape (v3.7.2) plugin MCODE was applied to identify significantly enriched subnetworks with the following parameters: degree cutoff of 2, node score cutoff of 0.2, k‐core of 2, and max depth of 100. For the RNA‐seq differentially expressed genes or those within the same PPI network module, KEGG and GO functional enrichment analyses were performed using the KOBAS database (http://kobas.cbi.pku.edu.cn/kobas3) (Bu et al. 2021), following the methodology described in section 2.3 RNA‐seq for pig mammary placodes. KEGG or gene ontology (GO) functional pathways with an FDR < 0.05 were considered significantly enriched.

### 2.5 Integration Analysis of ATAC‐Seq and RNA‐Seq

We performed an intersection analysis of genes annotated to differential OCRs (within 3 kb of accessible regions) (Wang et al. 2022) between Erhualian and Bamaxiang pigs by ATAC‐seq, and the differentially expressed genes identified between these two breeds by RNA‐seq. For the intersected genes from the two analyses, we converted gene read counts to TPM (Transcripts Per Million) values for expression visualization. Additionally, we used IGV (Integrative Genomics Viewer, v2.9.2) software to visualize the chromatin accessibility levels near the intersected genes.

### 2.6 Functional Verification Analysis of Candidate Genes at the Association Level

To preliminarily validate the functions of candidate genes, we performed a phenome‐wide association study (pheWAS) using the PigBiobank database (https://pigbiobank.farmgtex.org/) (Zeng et al. 2024). For each gene and the 298 traits examined (including 30 adaptation traits, 6 exterior traits, 57 health traits, 4 immune capacity traits, 2 litter traits, 60 meat and carcass traits, 31 production traits, and 108 reproduction traits), the PigBiobank database provides *p*‐values and cohort information for association analysis. We extracted these *p*‐values to generate a pheWAS Manhattan plot. Additionally, for the key candidate gene region near 48.80 Mb on SSC14, we used the PigBiobank database to check for significant GWAS statistics related to pig teat number.

Subsequently, this study collected 764 Suhuai pigs (hybrids of Western commercial Large White pigs and the Chinese indigenous Huai pig), 365 Sujiang pigs (hybrids of Western commercial Duroc pigs and the Chinese indigenous Jiangquhai pig), and 301 Sutai pigs (hybrids of Western commercial Duroc pigs and the Chinese indigenous Erhualian and Meishan pigs) for GWAS analysis of total teat number within a single population. Analyzed using the following model in the LDAK (v5.2):

y=u+Xb+Sc+Za+e

where ***y*** is a vector of phenotypes, ***u*** is the overall mean, ***b*** is the vector of fixed effects (birth year‐season and sex), ***c*** is the additive substitution effect, ***a*** is the vector of random additive genetic effects a ~ *N* (0, ***G**σ*_a_^2^), ***G*** is the genomic relationship matrix, *σ*_a_^2^ is the additive genetic variance, e is the vector of residual errors with e ~ *N* (0, ***I**σ*_e_^2^), where ***I*** is the identity matrix and *σ*_e_^2^ is the residual variance. ***X***, ***S***, and ***Z*** are the incidence matrices for ***b***, ***c***, and ***a***, respectively.

For the GWAS summary statistics of each individual population, we utilized METAL (v2011‐03‐25) to conduct meta‐analysis. Bonferroni multiple correction was applied to the GWAS *p*‐values to determine genome‐wide and suggestive significance thresholds. Using the R package LocusZoom, we generated a regional plot for the 48.80 Mb region on SSC14.

### 2.7 Linkage Disequilibrium Analysis

Linkage disequilibrium (LD) analysis was conducted for a 500 kb region surrounding the 48.80 Mb locus (±250 kb) in Erhualian and Bama Xiang pigs. The extent of LD in each breed was estimated based on pairwise genotype correlation coefficients (*r* ^2^) using PLINK (v1.90) with the command: “r2 ‐ld‐window 9999 ‐ld‐window‐r2 0 ‐ld‐window‐kb 1000”. Inter‐SNP distances (kb) were categorized into the following bins: 0–1, 1–3, 3–6, 6–9, 9–15, 15–30, 30–40, 40–60, 60–80, 80–100, 100–150, 150–200, 200–250, 250–300, 300–500. The average *r* ^2^ values for each distance bin were calculated and plotted as a function of increasing inter‐SNP distance.

### 2.8 Fluorescence In Situ Hybridization

The expression sites of the *ENSSSCG00000031037* gene mRNA in mammary placodes were validated using fluorescence in situ hybridization (FISH). We first prepared paraffin sections of 26‐day‐old Erhualian pig embryos fixed with a fixative, making serial sections parallel to the body axis. The sections containing the stratified epithelial structure of the mammary placodes were used for FISH experiments. Probes were supplied by Wuhan Xavier Biotechnology Co. The paraffin sections were deparaffinized, hydrated, washed, dehydrated with 70%, 85%, and 95% ethanol, and air‐dried. The probe mixture was added, denatured at 83°C for 5 min, and then hybridized at 42°C for 16 h. Sections were placed in 50% formamide/2× SSC to remove the coverslip, followed by washes in 50% formamide/2× SSC for 5 min, 2× SSC for 10 min, and 0.1% NP‐40/2× SSC for 3 min. They were rinsed in 70% ethanol for 4 min, air‐dried, counterstained with DAPI, mounted, and observed under a fluorescence microscope after being kept in the dark for 6 min.

### 2.9 Selection Signal Analysis

We extracted Erhualian pigs (*N* = 24) and Bamaxiang pigs (*N* = 21) resequencing data from the genotype‐imputed reference populations from the PigGTEx project (Teng et al. 2024) for selection signal detection. For the genotype data of these 45 samples, we used the PLINK (v1.90) for data quality control, excluding SNP loci with a MAF less than 0.05 and genotype missing rate greater than 0.1. First, we estimated the fixation index for each SNP between Erhualian pigs and Bamaxiang pigs using VCFtools (v0.1.17). The analysis was conducted with a 10 kb window size and a 5 kb step size. Genomic regions within the top 5% of Fst values were considered as significantly differentiated regions between the two breeds. Selscan (V1.2.0a) was used to carry out the integrated Haplotype Score (iHS) and number of Segregating sites by Length (nSL) analyses (Szpiech and Hernandez 2014) for Erhualian and Bamaxiang pigs separately, and performed an Cross‐Population Extended Haplotype Homozygosity (XP‐EHH) analysis (Szpiech and Hernandez 2014) for the combined Erhualian and Bamaxiang populations. The output values from the three analyses were separately normalized and converted to absolute values. Loci with values exceeding a threshold of 2 were considered as significantly selected sites. We performed Tajima's *D* (Eckshtain‐Levi et al. 2018) analysis for Erhualian and Bamaxiang pigs using VCFtools (v0.1.17) with the “‐‐TajimaD” option, employing a 10 kb window and 5 kb step size for the analysis. Genomic windows within the top 5% of Tajima's *D* values were considered as significantly subjected to balancing selection.

## 3 Results

### 3.1 Chromatin Accessibility Region Characterization in Pig Mammary Placodes

We first assessed the ATAC‐seq sequencing quality of the mammary placodes from 26‐day‐old pig embryos. PCR amplification of the *SRY* gene in three Erhualian and three Bamaxiang pig samples confirmed that each breed had one male pig and two female pigs, respectively. The Erhualian and Bamaxiang embryonic samples included both male and female individuals, effectively minimizing potential sex‐related biases in the analysis. The results showed (Figure 1B,C, Figure S1) that for the six samples, the nonredundant fraction (NRF), PCR Bottlenecking Coefficient 1 (PCB1), and PCR Bottlenecking Coefficient 2 (PCB2) values were 0.94 ± 0.02, 0.95 ± 0.02, and 23.84 ± 11.93, respectively. The NRF value represents the ratio of uniquely mapped reads to the total number of reads in ATAC‐seq data. A higher NRF value indicates a greater proportion of unique reads, suggesting better data quality. PCB1 and PCB2 values evaluate the effectiveness of PCR amplification. Generally, NRF > 0.9, PBC1 > 0.9, and PBC2 > 3 are required (Zhu et al. 2023), and all these indicators met the criteria, indicating good library sequencing consistency and quality. We analyzed the read lengths of the ATAC‐seq data for these six samples (Figure 1D, Figure S2), observing enrichment at 100 bp, suggesting that most of the insert fragments were around 100 bp, consistent with expected results (Zhang, Liu, et al. 2023; Zhu et al. 2023).

**FIGURE 2:**
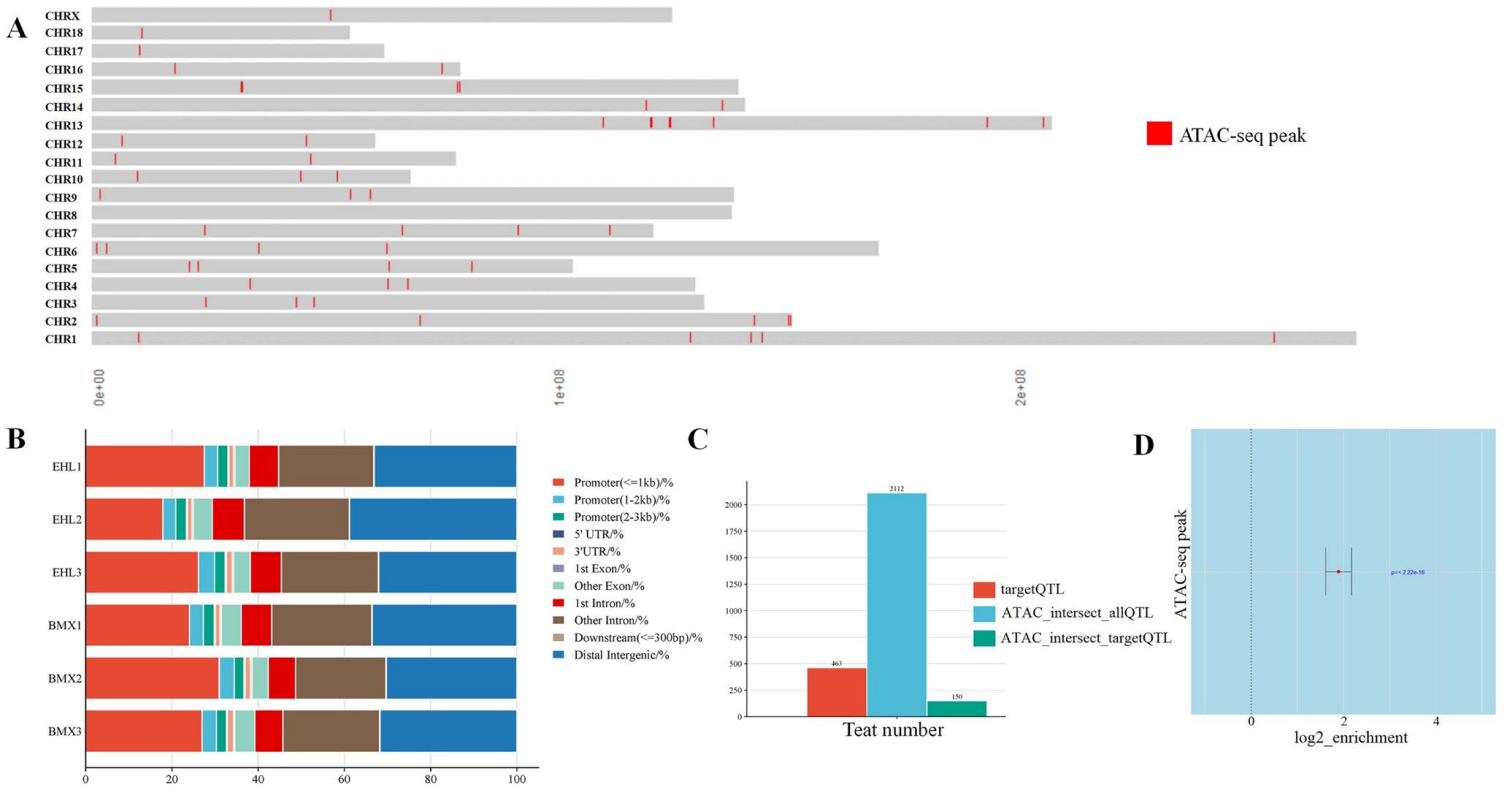
Distribution and functional annotation of chromatin accessibility regions in pig mammary placodes. (A) Genomic distribution of chromatin accessibility regions in pig mammary placodes, with red boxes indicating regions enriched in chromatin accessibility. (B) Annotation results of chromatin accessibility regions in genomic functional areas. Genomic functional regions are classified into 11 categories, including Promoter (≤ 1 kb), Promoter (1–2 kb), Promoter (2–3 kb), 5′ UTR, 3′ UTR, 1st Exon, Other Exon, 1st Intron, Other Intron, Downstream (≤ 300 bp), and Distal Intergenic. (C) Annotation results of chromatin accessibility regions in relation to the Pig QTL database. Target QTL refers to the 463 QTL intervals related to teat number reported in the Pig QTL database. ATAC_intersect_allQTL indicates the number of accessible regions intersecting with all QTL intervals in the Pig QTL database. ATAC_intersect_targetQTL indicates the number of intersections between accessible regions and teat number‐related QTL intervals in the Pig QTL database. (D) Enrichment results of chromatin accessibility regions with teat number‐related QTL intervals in the Pig QTL database. The length of the bars represents the 95% confidence interval.

In this study, a total of 30,806 peaks were identified across the six samples, with each peak corresponding to an OCR. The OCRs were significantly enriched near the transcription start site (TSS) of genes (Figure 1E, Figure S3). Furthermore, the OCRs of the mammary placodes were widely distributed across autosomes 1–18 and the X chromosome, indicating a high level of openness in the embryonic mammary placodes (Figure 2A). We conducted a preliminary functional annotation of these OCRs. Firstly, by annotating the identified OCRs in relation to the functional regions of the pig genome, we found that the majority of OCRs were located in distal intergenic regions, accounting for approximately 33.25% ± 2.95% of the total OCRs. This indicates that intergenic regions are crucial for gene expression regulation. Secondly, OCRs located in promoter regions constituted the second‐highest proportion, with OCRs within the 3 kb promoter range comprising 31.31% ± 4.47%. Compared to intergenic regions, promoter regions are relatively scarce across the genome, thus exhibiting a higher pattern of OCR enrichment. The proportion of OCRs subsequently decreased in intronic, exonic, and UTR regions (Figure 2B). To further annotate the function of OCRs, we performed an enrichment analysis using the Pig QTL database, focusing on 463 QTL intervals associated with pig teat number. We found that 150 QTL intervals related to teat number intersected with OCRs (Figure 2C). Fisher's exact test revealed that, compared to expected values, OCRs were significantly enriched in QTL intervals associated with pig teat number (Figure 2D). Additionally, there is overlap between OCRs and several top significant QTL intervals from the meta‐GWAS on total teat number traits in the PigBiobank database. These include the QTL interval on SSC7 containing *VRTN* gene and the QTL interval on SSC10 containing *FRMD4A* gene, both of which have been reported as candidate genes associated with teat number traits (Bovo et al. 2021; Klepinin et al. 2022). This result suggests that OCRs in embryonic mammary placodes may have a significant impact on variation in pig teat number.

### 3.2 Identification of Differential OCRs in Mammary Placodes Between Erhualian and Bamaxiang Pigs

**FIGURE 3:**
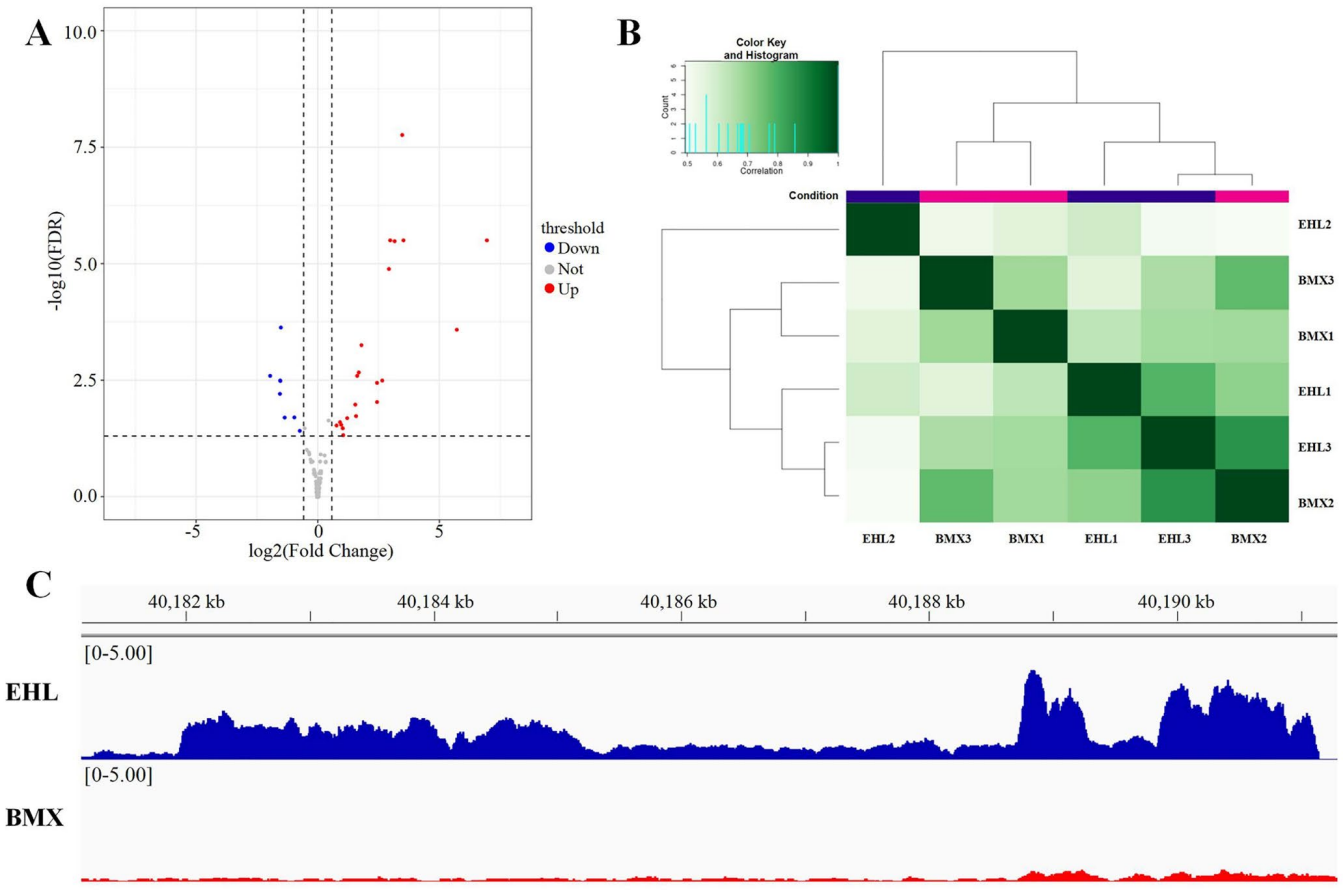
Identification of differential accessibility regions in mammary placodes of Erhualian and Bamaxiang Pigs. (A) Volcano plot of differential accessibility regions in mammary placodes between Erhualian and Bamaxiang pigs. Differential OCRs were identified with criteria of multiple testing using the false discovery rate (FDR) < 0.05. Using Bamaxiang pigs as the reference, red indicates significantly increased accessibility in Erhualian pigs, while blue indicates significantly decreased accessibility in Erhualian pigs. Gray indicate no significant differences between varieties. (B) Clustering heatmap of accessible regions in mammary placodes from three Erhualian pigs and three Bamaxiang pigs. EHL represents Erhualian pigs, and BMX represents Bamaxiang pigs. In the upper left corner, the color gradient from light green to dark green represents the increasing correlation coefficient among samples, indicating a progressive enhancement in the consistency of accessible regions among samples. The count denotes the distribution statistics of the correlation coefficients among samples. In the heatmap, the condition labels represent the Erhualian and Bamaxiang variety groupings. (C) Differential chromatin accessibility between Erhualian and Bamaxiang pigs between 48.10 Mb and 48.20 Mb on SSC5.

Given the significant difference in teat number between Erhualian and Bamaxiang pigs, where Erhualian pigs have an extremely high number of teats and Bamaxiang pigs have an extremely low number (Mo et al. 2022), this study compared the chromatin accessibility of mammary placodes from Erhualian pigs and Bamaxiang pigs to identify candidate differential OCRs with potential regulatory functions. The results showed a total of 30 differential peaks were identified, with 23 peaks exhibiting higher signals in Erhualian pigs and 7 peaks exhibiting higher signals in Bamaxiang pigs (Figure 3A, Table S1). This indicates that Erhualian pigs have higher accessibility in these differential peak regions.

However, considering the extreme difference in teat number phenotypes between Erhualian and Bamaxiang pigs and the total of 30,806 peaks, the number of differential peaks between the two breeds is relatively small (Guo et al. 2023; Nazzari et al. 2023; Zhang, Liu, et al. 2023), and clustering heatmaps based on peaks showed no clear population stratification between Erhualian and Bamaxiang pigs (Figure 3B), indicating a high level of conservation in the accessibility regions of mammary placodes across different pig breeds. Notably, we identified several highly significant differential peaks between 40.10 Mb and 40.20 Mb on SSC5 (Table S1). Visualization with IGV confirmed significant differences in accessibility in this region between Erhualian and Bamaxiang pigs (Figure 3C), validating the reliability of the identified differential peaks in this study.

For the 30 differential OCRs identified, we performed gene annotation within a 3 kb region and annotated seven protein‐coding genes, one lncRNA, and one miRNA (Table S2). These genes are important functional candidates. Focusing on the functions of these genes, the *AK6* gene is crucial for embryonic development (Bai et al. 2016) and is also associated with breast cancer formation (Klepinin et al. 2022). The *ABCG2* gene has been reported to be related to the proliferation of mammary epithelial cells (Wei et al. 2012).

We further analyzed the differential OCRs of mammary placodes between Erhualian and Bamaxiang pigs to identify enriched transcription factor motifs. The enriched transcription factors are key candidates that may contribute to the differences in teat number between Erhualian and Bamaxiang pigs. Only two transcription factor motifs were enriched (Table 1), namely *NEUROD2* and *OLIG2*. Previous studies have reported the role of *OLIG2* in the epithelial‐mesenchymal transition (EMT) process (Sailer et al. 2016). Additionally, *NEUROD2* has been associated with breast cancer formation (Lacle et al. 2015).

**TABLE 1:** Predicted binding motifs in the differential open chromatin region between Erhualian and Bamaxiang pigs.

Motifs	TF ID	*p*	*E*
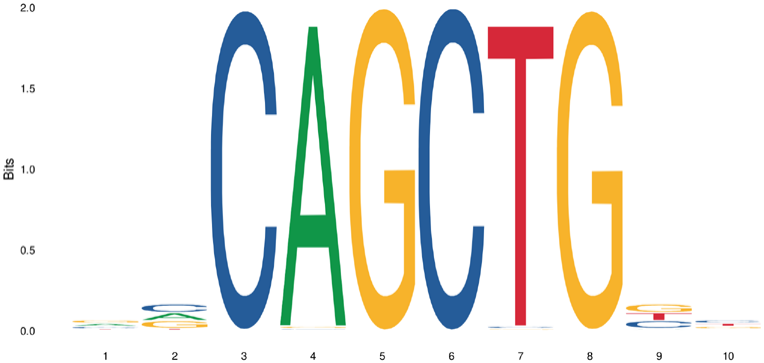	*NEUROD2*	1.04e‐6	8.74e‐4
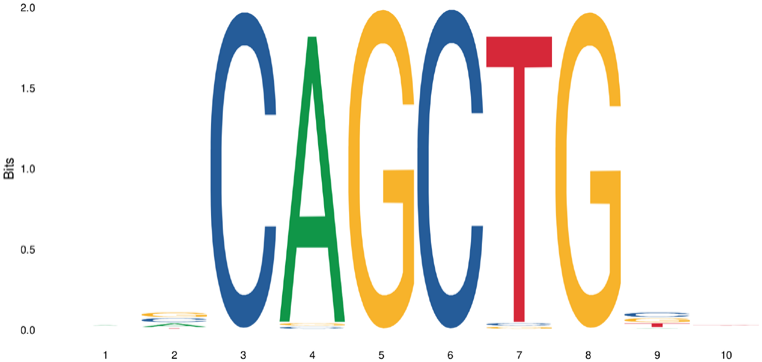	*OLIG2*	8.33e‐4	7.00e‐1

### 3.3 Identification of Differentially Expressed Gene in Mammary Placodes Between Erhualian and Bamaxiang Pigs

**FIGURE 4:**
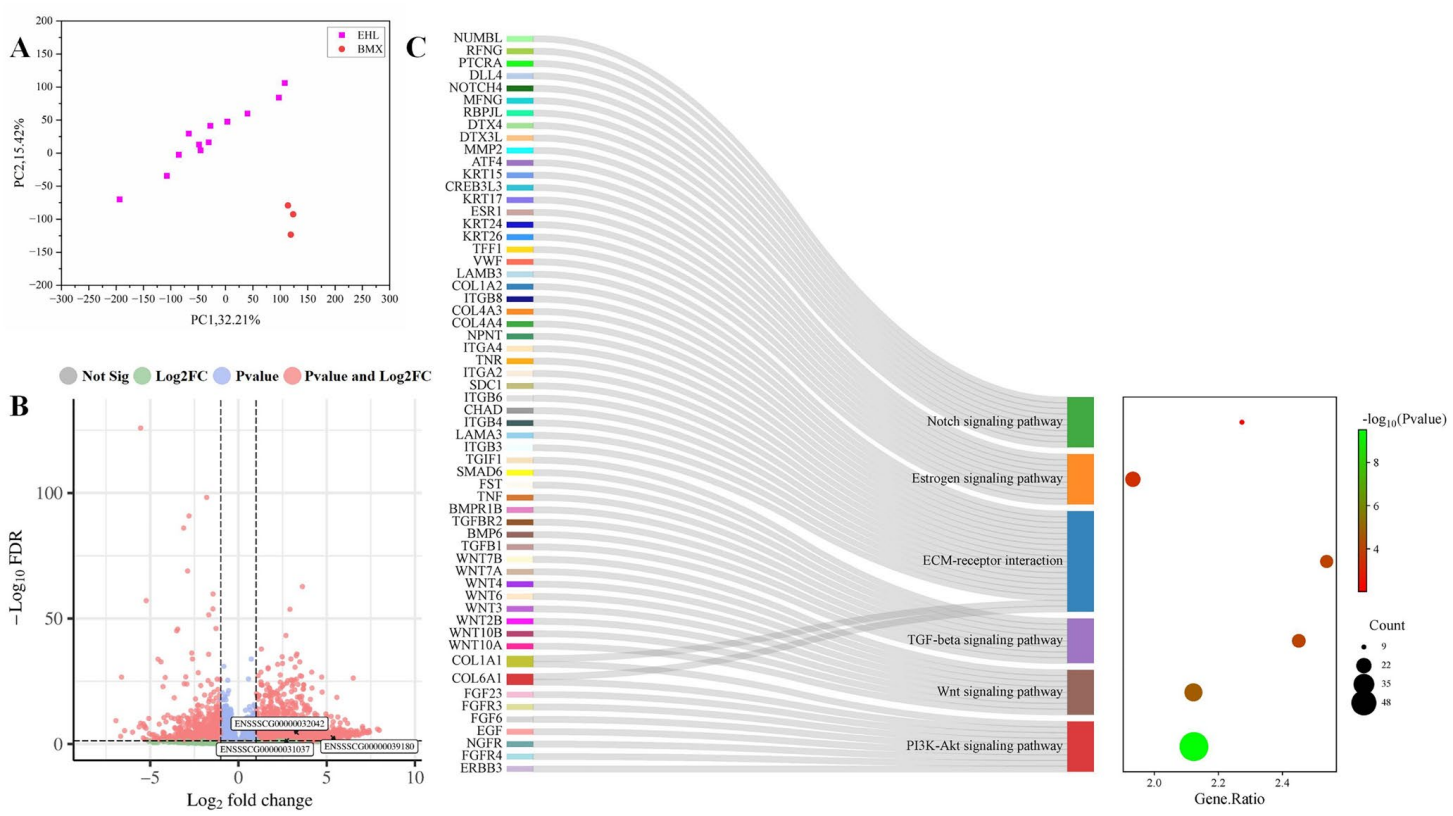
Identification of differentially expressed genes in mammary placodes between Erhualian and Bamaxiang pigs. (A) PCA plot of gene expression in mammary placodes from 12 Erhualian pig individuals and three Bamaxiang pig individuals. EHL represents Erhualian pigs, and BMX represents Bamaxiang pigs. (B) Volcano plot of differentially expressed genes in mammary placodes of Erhualian and Bamaxiang pigs. Not Sig indicates genes with no significant expression differences between breeds, defined by FDR ≥ 0.05 and log2FoldChange ≤ 1. Log2FC refers to genes with FDR > 0.05 but a log2FoldChange > 1. *p* value represents genes with FDR < 0.05 but log2FoldChange ≤ 1. *p* value and Log2FC refers to differentially expressed genes (DEGs) with FDR < 0.05 and log2FoldChange > 1. The white boxes highlight the three candidate genes, *ENSSSCG00000031037, ENSSSCG00000032042*, and *ENSSSCG00000039180*, showing their differential expression status among all genes. (C) KEGG signaling pathways related to the formation and development of mammary placodes enriched by differentially expressed genes between Erhualian and Bamaxiang pigs. Gene Ratio denotes the enrichment ratio, count represents the number of genes enriched in a specific pathway, and *p* value refers to the adjusted *p* value after multiple testing correction. The Sankey diagram on the left displays enriched pathways corresponding to key candidate genes related to mammary placodes development.

To identify differentially expressed genes in mammary placodes between Erhualian and Bamaxiang pigs, we selected mammary placodes from 12 Erhualian pig individuals and 3 Bamaxiang pig individuals for RNA‐seq analysis. PCR amplification of the *SRY* gene revealed that among the 12 Erhualian pig samples, 7 were male and 5 were female. In the 3 Bamaxiang pig samples, 1 was male and 2 were female. The Erhualian and Bamaxiang embryonic samples included both male and female individuals, ensuring a balanced representation of sexes in the analysis. Both the PCA plot and the clustering heatmap of the sequencing samples demonstrated clear population stratification between Erhualian and Bamaxiang pigs at the overall gene expression level (Figure 4A, Figure S4), indicating distinct gene expression patterns in mammary placodes between these two breeds. A total of 4432 differentially expressed genes were identified, with 3463 genes upregulated and 969 genes downregulated in Erhualian pigs compared to Bamaxiang pigs (Figure 4B). This includes important marker genes such as *VRTN*, *WNT10B, LEF1*, and *ERBB4* (Veltmaat et al. 2004; Zeng et al. 2024).

Kyoto Encyclopedia of Genes and Genomes functional enrichment analysis of these differentially expressed genes revealed several pathways crucial for the formation and development of mammary placodes in various species (Figure 4C, Table S3). Firstly, marker genes associated with mammary placode formation, such as *WNT10A*, *WNT10B*, and *WNT6* (Veltmaat et al. 2004; Zeng et al. 2024), were significantly enriched in the WNT signaling pathway (FDR = 1.89E‐05). The WNT signaling pathway has been reported to be involved in both mammary line formation and mammary placode formation (Boras‐Granic et al. 2006; Wysolmerski et al. 1998). The Notch signaling pathway was observed to be significantly enriched (FDR = 9.33E‐03). In mammary development, the Notch signaling pathway is reported to regulate interactions between epithelial and mesenchymal cells, playing a crucial role in ductal formation and differentiation (Buono et al. 2006). Among the differentially expressed genes enriched in the Notch signaling pathway, *NOTCH4*, as a member of the Notch receptor family, is expressed in mammary epithelial cells and is involved in the regulation of mammary gland function (Soriano et al. 2000). Estrogen signaling (FDR = 3.96–04) is vital for mammary development, regulating the proliferation, differentiation, and survival of mammary epithelial cells through estrogen receptor‐mediated signaling (Wan et al. 2022). Genes enriched in the estrogen signaling pathway include *ESR1*, a key regulator of this pathway, as well as *KRT17* and *KRT15*, which serve as marker genes for epithelial cells (Sartaj et al. 2017). In fact, the formation of mammary placodes partially results from epithelial‐mesenchymal interactions. We found that extracellular matrix‐related (ECM‐related) pathways, which influence epithelial signaling and mesenchymal cell differentiation (Drake and Franz‐Odendaal 2018; Maller et al. 2010), were significantly enriched (FDR = 2.56E‐05). Among the key genes enriched in this pathway, *ITGB4* was found to influence mammary placode formation by mediating the PTHrP pathway. Notably, *ITGB4*‐knockout mice were unable to generate mammary glands when transplanted into cleared fat pads (Li et al. 2015). The transforming growth factor beta (TGF‐beta) signaling pathway (FDR = 2.35E‐05) regulates the proliferation and differentiation of mammary epithelial cells during development, impacting mammary morphogenesis and functional maturation (Zhang, van der Zon, et al. 2023). Among the differentially expressed genes enriched in the pathway, *TGFB1* regulates mammary epithelial branching morphogenesis and extracellular matrix remodeling, while also inhibiting excessive ductal outgrowth (Vincent et al. 2003). *BMP6* was also enriched in the TGF‐beta signaling pathway. *BMP6* and other bone morphogenetic protein (BMP) family genes are crucial for mammary placode formation and early development, contributing to the maintenance of the mammary stem cell niche and potentially influencing placode formation (Katoh 2017). As a BMP signaling inhibitor, the differentially expressed gene *SAMD6* may alter mammary epithelial cell function (Gatza et al. 2014). The Phosphoinositide 3‐kinase—Protein Kinase B (PI3K‐Akt) signaling pathway (FDR = 6.92E‐12) is involved in survival signaling in mammary epithelial cells, essential for mammary tissue growth and maintenance (Maharati and Moghbeli 2023). FGF receptor genes, including *FGFR3* and *FGFR4*, are enriched in this pathway. Studies have shown that FGF receptor genes are expressed in the ectodermal epithelium and mammary placode (Mailleux et al. 2002). Additionally, *ERBB3*, a member of the epidermal growth factor receptor (EGFR/HER) family, promotes mammary epithelial cell proliferation and survival by activating the PI3K/AKT and mitogen‐activated protein kinase (MAPK) signaling pathways (Samant and Sylvester 2006). These results suggest that the differentially expressed genes in the mammary placodes between Erhualian and Bamaxiang pigs are potentially involved in several critical pathways of mammary placodes formation and development, making them important candidate functional genes.

### 3.4 Construction of Coexpression Networks of Differentially Expressed Genes and Transcription Factors Enriched in Differential OCRs Between Erhualian and Bamaxiang Pigs

**FIGURE 5:**
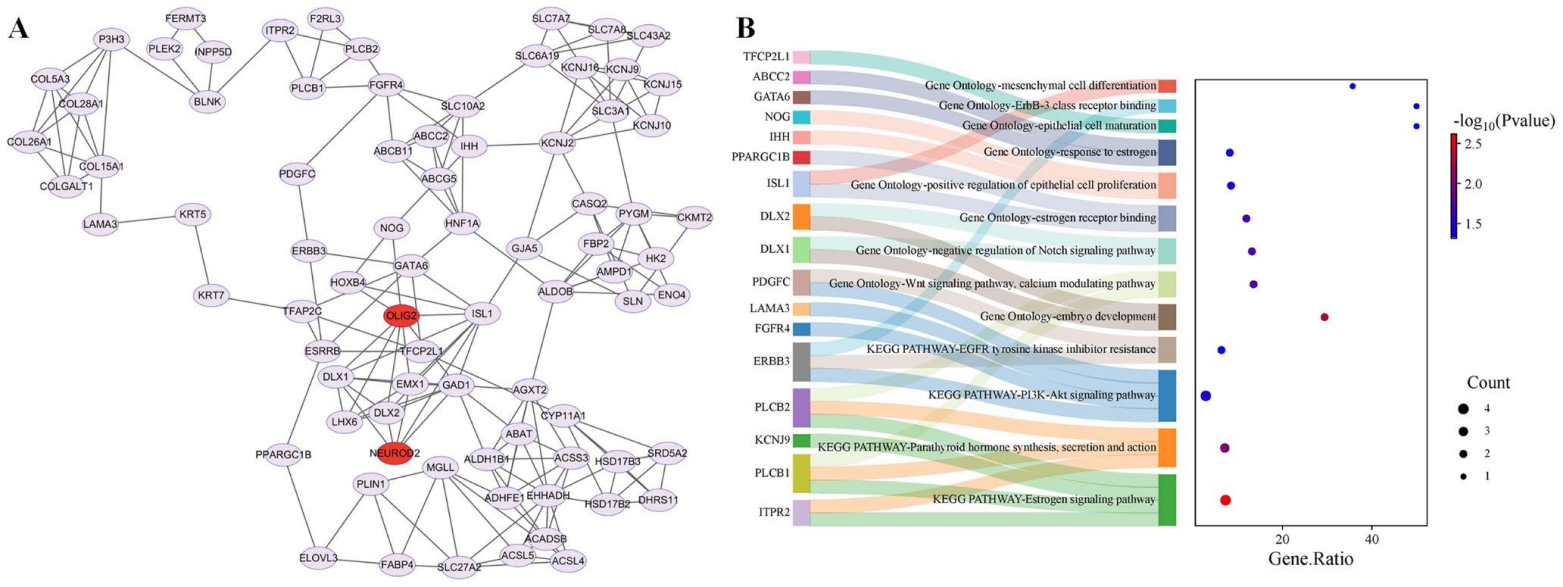
Construction of PPI network for transcription factors enriched in differential chromatin accessibility regions and differentially expressed genes between Erhualian and Bamaxiang pigs. (A) Subnetwork constructed based on the MCODE module in Cytoscape, identifying differentially expressed genes with significant interactions with key transcription factors *OLIG2* and *NEUROD2*. (B) KEGG and GO pathways related to the formation and development of mammary placodes enriched by differentially expressed genes with significant interactions with *OLIG2* and *NEUROD2*. Gene Ratio denotes the enrichment ratio, count represents the number of genes enriched in a specific pathway, and *p* value refers to the adjusted *p* value after multiple testing correction. The Sankey diagram on the left displays enriched pathways corresponding to key candidate genes related to mammary placodes development.

In the comparison between Erhualian and Bamaxiang pig breeds, two transcription factors, *NEUROD2* and *OLIG2*, were enriched in differential OCRs. These transcription factors are important candidates influencing the variation in the number of pig teats (Lacle et al. 2015; Sailer et al. 2016). To determine the impact of these transcription factors on gene expression between these two pig breeds, we integrated differentially expressed genes with the aforementioned transcription factors to construct a PPI network. We discovered that *NEUROD2* and *OLIG2*, along with a part of differentially expressed genes, form a closely interacting submodule (Figure 5A). It is hypothesized that the differential expression of these genes might be partially due to differences in the binding patterns of *NEUROD2* and *OLIG2* in differential OCRs.

Functional annotation of genes that interact closely with the transcription factors *NEUROD2* and *OLIG2* through KEGG and GO enrichment analyses revealed that these genes are significantly enriched in pathways related to the formation of mammary placodes' epithelial cells (Figure 5B, Table S4). Among these pathways is the Estrogen signaling pathway (FDR = 2.41E‐03). The key gene *PLCB1*, which is enriched in this pathway, has been reported to be highly expressed in bovine mammary epithelial cells and to influence calving performance in cattle (Feng et al. 2024). The *ERBB3* (Samant and Sylvester 2006) and *FGFR4* (Mailleux et al. 2002) genes are enriched in the PI3K‐Akt signaling pathway (FDR = 6.21E‐03) and are also involved in the positive regulation of epithelial cell proliferation (Maharati and Moghbeli 2023; Wan et al. 2022). Additionally, mammary placode mesenchymal cells, as a crucial supportive cell type for mammary placode epithelium cell, play a role in mammary placode formation. Among the closely interacting genes mentioned above, we identified several that are involved in key pathways related to the generation of mammary mesenchymal cells. Parathyroid hormone synthesis, secretion, and action is an important pathway influencing the formation of mammary mesenchyme. The receptor for parathyroid hormone‐related protein, *PTH1R*, is expressed in the mesenchyme of the developing mammary placode, promoting mammary development (Wysolmerski et al. 1998). In addition, the mesenchymal cell differentiation pathway (Spina and Cowin 2021) is associated with mesenchyme, where the key gene *ISL1* has been reported to participate in epithelial to mesenchymal transition (EMT). EMT plays a crucial role in the process of mammary placode formation (Choi et al. 2021). We also identified the embryo development pathway, and the genes enriched in this pathway may exert an influence on embryonic development. These findings suggest that the differentially expressed genes closely interacting with *NEUROD2* and *OLIG2* may have a certain influence on the formation and development of the mammary placodes.

### 3.5 Integrated Analysis of ATAC‐Seq and RNA‐Seq Between Erhualian and Bamaxiang Pigs

**FIGURE 6:**
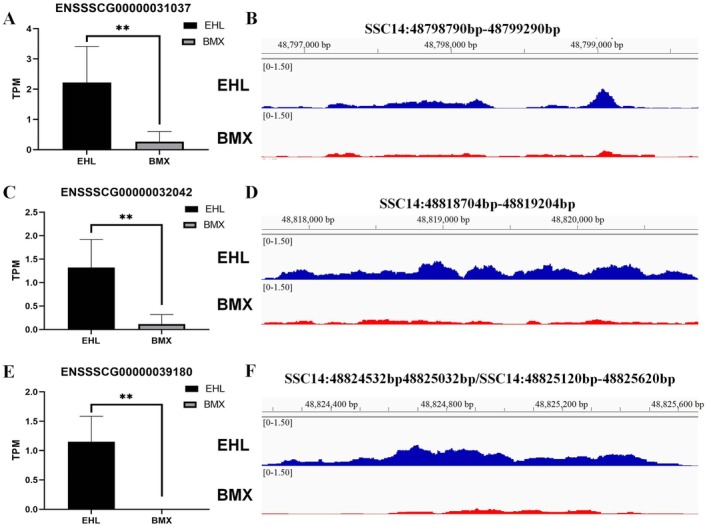
Integrated analysis of ATAC‐seq and RNA‐seq in pig mammary placodes. Genes annotated from differential chromatin accessibility regions and differentially expressed genes between Erhualian and Bamaxiang pigs identified three intersecting protein‐coding genes: *ENSSSCG00000031037*, *ENSSSCG00000032042*, and *ENSSSCG00000039180*. (A, B) Differential expression and chromatin accessibility for the *ENSSSCG00000031037* gene between the two breeds. **Represents *p* value < 0.01. (C, D) Differential expression and chromatin accessibility for the *ENSSSCG00000032042* gene between the two breeds. (E, F) Differential expression and chromatin accessibility for the *ENSSSCG00000039180* gene between the two breeds.

To determine whether differential gene expression between Erhualian and Bamaxiang pigs may result from differences in chromatin accessibility near genes, ultimately leading to phenotypic differences between breeds, we conducted an intersection analysis of candidate genes annotated in differential OCRs with differentially expressed genes between the breeds. The results identified three protein‐coding genes located in differential OCRs, where the chromatin accessibility in the mammary placodes of Erhualian pigs was significantly higher than that in Bamaxiang pigs. In addition, gene expression levels in Erhualian pigs were also significantly higher than in Bamaxiang pigs. These genes are *ENSSSCG00000031037* (FDR = 3.87E‐03, log2FoldChange = 2.87) (Figure 6A,B), *ENSSSCG00000032042* (FDR = 2.78E‐04, log2FoldChange = 3.43) (Figure 6C,D), and *ENSSSCG00000039180* (FDR = 2.58E‐04, log2FoldChange = 5.22) (Figure 6E,F). All three genes are located near the 48.80 MB region on SSC14 and are adjacent to each other.

**FIGURE 7:**
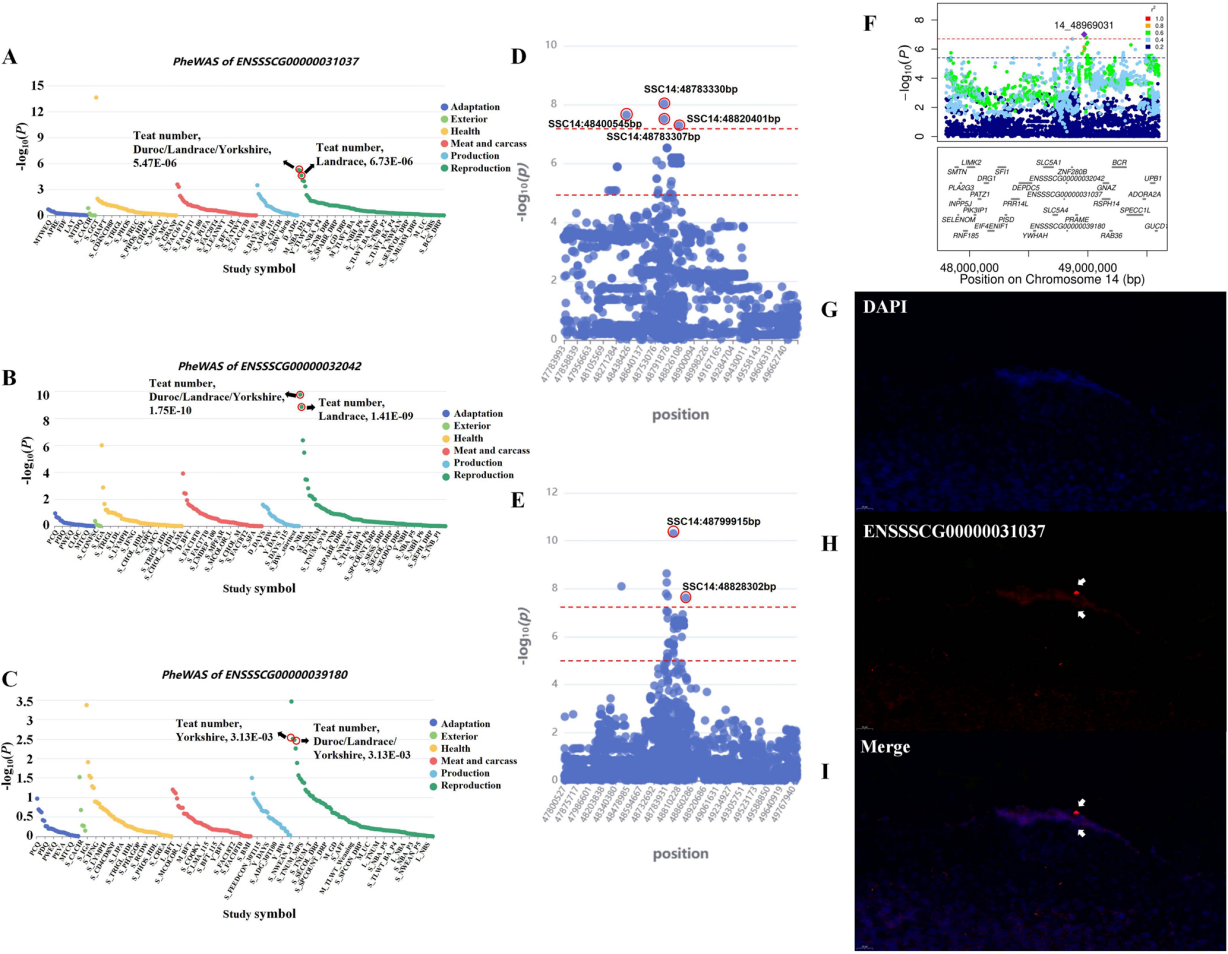
Functional annotation of candidate genes. (A–C) The pheWAS Manhattan plots for *ENSSSCG00000031037*, *ENSSSCG00000032042*, and *ENSSSCG00000039180*, respectively. Red circles indicate significant loci associated with teat number across different pig breeds. (D) Local zoom plot of GWAS results for teat number in Landrace pigs near the 48.80 Mb region on SSC14. The red dashed lines indicate the significance thresholds at the chromosome and genome levels. (E) Local zoom plot of meta‐GWAS results for teat number in Duroc/Landrace/Yorkshire mixed populations at the 48.80 Mb region on SSC14. The red dashed lines indicate the significance thresholds at the chromosome and genome levels. (F) Local zoom plot of meta‐GWAS results for teat number in the Chinese‐Western hybrid pig breeds Suhuai, Sujiang, and Sutai pigs at the 48.80 Mb region on SSC14. The dashed lines indicate the significance thresholds at the chromosome and genome levels. (G–I) FISH results for *ENSSSCG00000031037* gene. From top to bottom are DAPI staining, fluorescent staining of *ENSSSCG00000031037* gene, and the merged result. The *ENSSSCG00000031037* gene is specifically expressed in the epithelial cells of mammary placodes near the ectoderm.

Currently, there is limited functional research on these three genes. To preliminarily determine their functions, we performed pheWAS using the PigBiobank database (Zeng et al. 2024). The analysis revealed significant associations between these genes and the teat number trait in different pig populations. Specifically, the gene *ENSSSCG00000031037* is significantly associated with the teat number trait in the Duroc/Landrace/Yorkshire mixed population (*p* value = 5.47E‐06) and the Landrace population (*p* value = 6.73E‐06) (Figure 7A). The gene *ENSSSCG00000032042* is also significantly associated with the teat number trait in the Duroc/Landrace/Yorkshire mixed population (*p* value = 1.75E‐10) and the Landrace population (*p* value = 1.41E‐09) (Figure 7B). The gene *ENSSSCG00000039180* is significantly associated with the teat number trait in the Duroc/Landrace/Yorkshire mixed population (*p* value = 3.13E‐03) and the Yorkshire population (*p* value = 3.13E‐03) (Figure 7C). These findings suggest that these three genes could not only influence the variation in teat number between Erhualian and Bamaxiang pigs but may also affect teat number traits in other pig breeds, including Western commercial pigs.

To further validate the function of the aforementioned genes at the association level, we annotated the regions where these genes are located using the GWAS statistics from the PigGTEx project (Teng et al. 2024), focusing on traits related to pig teat number. The results indicated that in the GWAS analysis of teat number trait in the Landrace population (*N* = 18,736), there is a genome‐wide significant signal near the 48.80 MB region on SSC14, with the most significant locus at SSC14:48783330 bp (*p* value = 9.29E‐09) (Figure 7D). Similarly, in the meta‐GWAS analysis of the Duroc/Landrace/Yorkshire mixed population (*N* = 118,477), a genome‐wide significant signal is present in the same region, with the most significant locus at SSC14:48799915 bp (*p* value = 4.89E‐11) (Figure 7E). To further elucidate the impact of the 48.80 Mb region on SSC14 on total teat number variation in Chinese indigenous pig breeds, including Erhualian pigs, we selected representative hybrid pig breeds, including Suhuai pigs, Sujiang pigs, and Sutai pigs, as the study subjects. These hybrid breeds' maternal lineages are Chinese indigenous pig breeds, such as Erhualian pigs, Huai pigs, and Jiangquhai pigs. Meta analysis of the GWAS summary statistics from individual breeds revealed a genome‐wide significant signal in this region, with the top SNP located at SSC14: 48969031 bp (*p* value = 9.77E‐08) (Figure 7F).

Linkage disequilibrium analysis of the 500 kb region (250 kb upstream and downstream of the 48.80 Mb region) in Erhualian and Bamaxiang pigs demonstrated that the LD level (*r* ^2^ = 0.3) in this region exceeded 500 kb for both breeds (Figure S5). This contrasts with the reported genome‐wide LD levels, where the LD decay (*r* ^2^ = 0.3) was 9.5 kb for Erhualian pigs and 12.5 kb for Bamaxiang pigs (Liu et al. 2020). Combined with pheWAS and GWAS annotation results for teat number, these findings suggest that Erhualian and Bamaxiang pigs may have undergone strong selection for teat number traits, leading to increased LD in this localized genomic region. These findings indicate that this genome region could significantly influences teat number variation across different pig breeds, including Western commercial breeds and Chinese indigenous breeds such as Erhualian pigs.

To further confirm the potential role of the three key candidate genes located in this region in affecting pig teat number variation, we selected *ENSSSCG00000031037* as candidate gene and conducted a FISH experiment on embryonic paraffin sections of Erhualian pigs to further validate the mRNA expression location. The results demonstrated that, compared to the background DAPI staining, *ENSSSCG00000031037* is expressed to some extent in the multilayered epithelial structure of the mammary placodes, particularly in epithelial cells near the ectoderm, where mRNA expression is relatively high and specific. There is no expression in the mesenchymal cells surrounding the mammary placodes (Figure 7G–I). These results indicate that the *ENSSSCG00000031037* gene is specifically expressed at the mRNA level in mammary placodes. Together with the results from multi‐omics analyses, genes like *ENSSSCG00000031037* could be key candidates influencing teat number variation between pig breeds, including Erhualian and Bamaxiang pigs.

### 3.6 Strong Selection Near the 48.80 Mb Region on SSC14 in Erhualian Pigs

Based on the ATAC‐seq and RNA‐seq analysis of mammary placodes between Erhualian and Bamaxiang pigs, three genes near the 48.80 Mb region on SSC14 could be important candidate genes explaining the differences in teat number between the breeds. The pheWAS and GWAS statistics further confirmed that this region, along with these three genes, is associated with teat number phenotypic variation in several Western commercial pig breeds. Moreover, LD analysis revealed that this region exhibits high LD degree in both Erhualian and Bamaxiang pig populations, with *r* ^2^_0.3_ exceeding 500 kb. To further investigate the genomic reasons behind the differences in teat number between Erhualian and Bamaxiang pigs, particularly the cause of the higher teat number in Erhualian pigs, we conducted various selection signal analyses on these two breeds. These analyses aimed to determine whether this region has undergone selection in Erhualian pigs, potentially leading to the breed‐specific high teat number phenotype.

**FIGURE 8:**
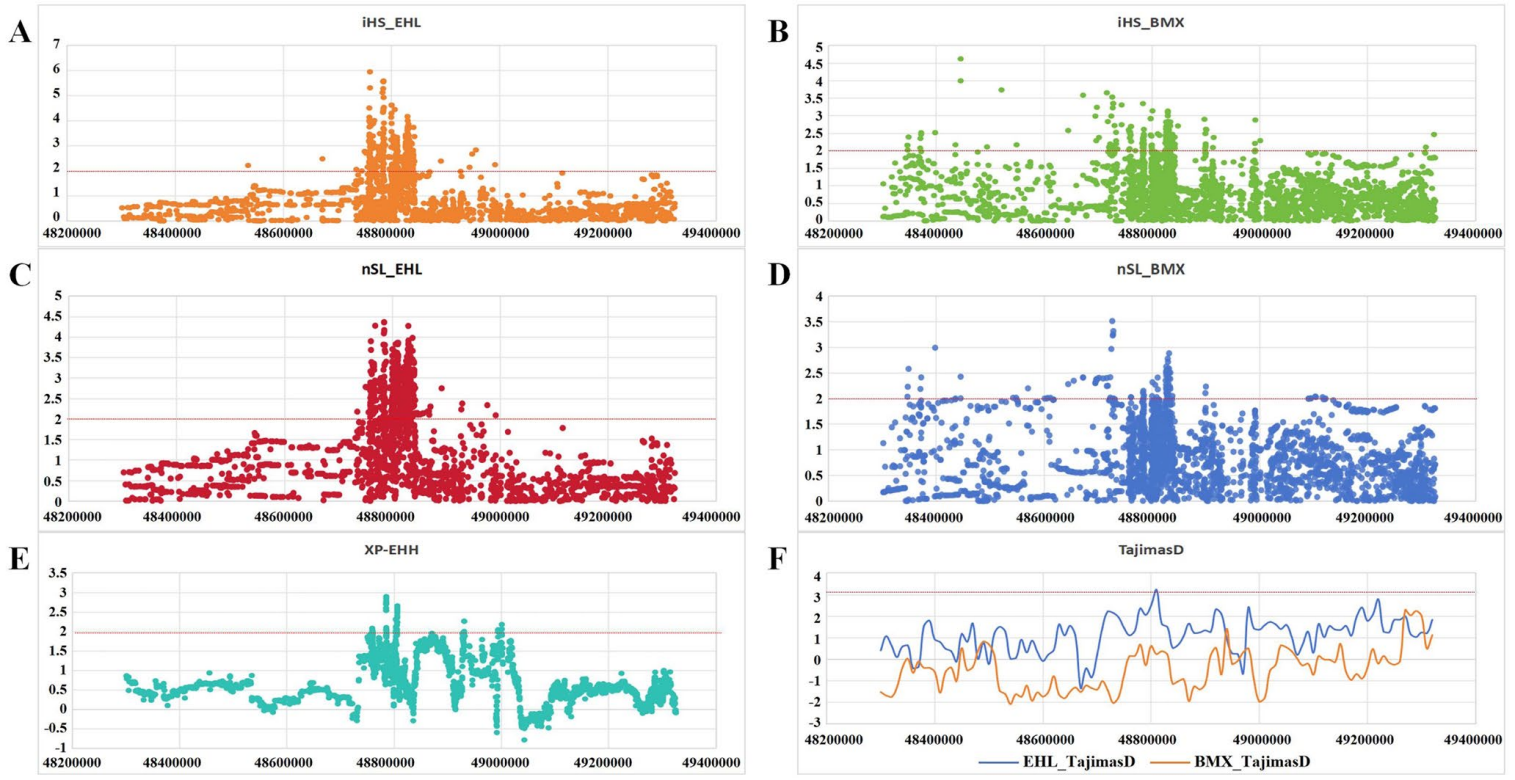
Detection of selection signals in key candidate region at 48.80 Mb on SSC14 in Erhualian Pigs. (A, B) iHS analysis for Erhualian pigs and Bamaxiang pigs, respectively. The red dashed line indicates the significance threshold (iHS value = 2). (C, D) nSL analysis for Erhualian pigs and Bamaxiang pigs, respectively. The red dashed line indicates the significance threshold (nSL value = 2). (E) XP‐EHH analysis between Erhualian and Bamaxiang pigs. Positive values indicate loci under selection in Erhualian pigs. The red dashed line indicates the significance threshold (XP‐EHH value = 2). (F) Tajima's *D* analysis for Erhualian and Bamaxiang pigs. Positive values indicate stronger balancing selection in the population. The red dashed line indicates the significance threshold (top 5%, Tajima's *D* value = 3.10).

Firstly, we performed Fst analysis based on allele frequency differences between Erhualian pigs and Bamaxiang pigs. The results showed that this region did not exhibit significant differentiation signals between the two breeds (Figure S6). Subsequently, we conducted analyses using selection signal methods based on LD. The results revealed that the iHS analysis identified a significant selection signal in the 48.80 Mb region in Erhualian pigs, with the highest iHS value being 5.96 (Figure 8A). In contrast, while Bamaxiang pigs also exhibited a selection signal in this region (iHS > 2), the signal strength was not as pronounced as in Erhualian pigs (Figure 8B). Similarly, nSL analysis revealed that Erhualian pigs exhibited a significant selection signal in the 48.80 Mb region, with a maximum nSL value of 4.36 (Figure 8C). In contrast, while Bamaxiang pigs also showed a selection signal (nSL > 2), the selection intensity was weaker compared to that in Erhualian pigs (Figure 8D). Combined with the Fst analysis, which indicated no significant differentiation signals between the two breeds, these findings suggested that both Erhualian and Bamaxiang pigs had undergone selection in the same direction in this region, but Erhualian pigs had experienced relatively stronger selection pressure. These results are consistent with the observation that LD level in Erhualian pigs is slightly higher than that in Bamaxiang pigs. The XP‐EHH results indicated that Erhualian pigs have undergone stronger selection in this region compared to Bamaxiang pigs (Figure 8E), possibly leading to the specific phenotypic trait of higher teat numbers in Erhualian pigs. The Tajima's *D* results also demonstrated a significant positive selection signal in the 48.80 Mb region on SSC14 in Erhualian pigs (top 5%, Tajima's *D* value = 3.10) (Figure 8F), further indicating strong selection in this region, primarily through balancing selection.

## 4 Discussion

In fact, the critical time point for determining the number of pig teats is embryonic day 26, when the mammary line further develops into the mammary placodes, which subsequently develops into the mammary bud. The mammary placodes serve as the precursor tissue for the teat (Chu et al. 2004; Mailleux et al. 2002; Sakai et al. 2019; Spina and Cowin 2021). Theoretically, each mammary placodes has the potential to develop into a teat. However, current research on pig teat numbers has not focused on the mammary placodes. Based on this, the present study is the first known study to conduct ATAC‐seq on pig mammary placodes.

To systematically identify the overall patterns of chromatin accessibility in mammary placodes, we selected three samples each from Erhualian pigs and Bamaxiang pigs, which exhibit significant phenotypic differences in teat number (China National Committee on Animal Genetic Resources 2011). Integrating the chromatin accessibility results from these six samples, we found that the chromatin accessibility distribution of this tissue is extensive, covering a wide range of areas on autosomes 1–18 and the X chromosome. The chromatin accessibility regions are significantly enriched in QTL intervals related to teat number in pigs and overlap with key candidate genes affecting teat number variation, such as the *VRTN* gene (Zeng et al. 2024). These results provide valuable insights for the epigenetic annotation of pig mammary placodes and lay a foundation for future studies on functional genes (Perrin et al. 2021; Quan et al. 2024; Zhu et al. 2023).

Previous studies have found that chromatin accessibility is broadly distributed in various tissues of early embryos across different species. This openness allows transcription factors and other regulatory proteins easier access to DNA, thereby promoting gene expression. During embryonic development, cells need to rapidly divide and differentiate to form different tissues and organs, and many genes are actively expressed at this stage to support these vital biological processes (Wu et al. 2016; Xu et al. 2024). Our study found that the chromatin accessibility regions of mammary placodes are broadly distributed across genomic regions, which aligns with expected results. This suggests that gene expression in this tissue may be subject to relatively extensive and complex regulation, with numerous genes potentially playing roles in the development of mammary placodes (Cusanovich et al. 2014).

However, we found that even in pig breeds with extreme phenotypes, such as Erhualian and Bamaxiang pigs, only 30 out of 30,806 chromatin accessible regions exhibited differences between the breeds. Clustering analysis of the chromatin accessible regions revealed no population stratification between Erhualian and Bamaxiang pigs, indicating that the chromatin accessibility of mammary placodes may be highly conserved across different pig breeds. This conservation may be due to the critical developmental stage, such as early embryonic development, where important regulatory genes and their networks and elements are often highly conserved (Ming et al. 2021; Zhu et al. 2024). In early embryonic tissues, chromatin accessibility exhibits high conservation across different breeds and even species. Studies have reported that during organogenesis in mice and humans, chromatin accessibility undergoes highly dynamic changes within specific developmental windows, which align with the temporal sequence of organ primordium formation. For example, the timing of regulatory element opening in organs such as the lungs and stomach shows a conserved sequence across species (Pijuan‐Sala et al. 2020; Smith et al. 2022; Sun et al. 2024). This suggests that chromatin states in early embryos are tightly regulated, with a relatively stable fundamental framework. This could explain the limited divergence in chromatin accessibility observed in mammary placodes between Erhualian and Bamaxiang pigs. Therefore, studies on the chromatin accessibility of mammary placodes using a single breed can often represent the overall patterns of chromatin accessibility in this tissue to some extent. Mammary placodes contain a variety of cell types, mainly including epithelial and mesenchymal cells, and chromatin accessibility may vary among different cell types (Cai et al. 2023; Wang et al. 2023). The next plan is to perform single‐cell ATAC‐seq on mammary placodes to further analyze the accessibility patterns of important cell types such as epithelial and mesenchymal cells. This will help to specifically elucidate the developmental patterns of these two cell types and their impact on the formation and development of mammary placodes.

Unlike the results of chromatin accessibility, there are a significant number of differentially expressed genes between Erhualian and Bamaxiang pigs at the transcriptomic level of the mammary placodes, totaling 4432 genes. The gene expression patterns exhibit clear stratification between the breeds, indicating that the overall patterns of gene expression in the mammary placodes differ between the two pig breeds. This inter‐breed variation may arise from changes in the DNA sequence within accessible chromatin regions, leading to alterations in regulatory element binding patterns, rather than differences in chromatin accessibility patterns between breeds. Additionally, other transcriptional and epigenetic regulatory mechanisms may also play a role. Posttranscriptional modifications, including RNA splicing, RNA stability, and posttranslational regulation, are likely to be critical in early embryonic development, potentially leading to discrepancies between gene expression levels. Studies have shown that in mouse and human early embryos, m6A methylation dynamically modifies mRNA and noncoding RNA, precisely regulating embryonic genome activation and cell fate determination (Gao et al. 2018). In terms of epigenetic transitions, in addition to chromatin accessibility, other epigenetic mechanisms such as DNA methylation (Lu et al. 2021) and histone modifications (Liu et al. 2016) may also play a significant role in regulating gene expression. These mechanisms may exhibit more pronounced differences across different breeds, thereby driving substantial gene expression changes.

Among these differentially expressed genes, several important functional genes have been identified, such as *VRTN*, *WNT10B*, *WNT6*, *LEF1*, and *ERBB4* (Boras‐Granic et al. 2006; Howard et al. 2005; Veltmaat et al. 2004; Zeng et al. 2024), all of which have been reported as key regulators influencing the formation and development of mammary placodes across different species, or as candidate genes significantly associated with teat number variation in pigs. This suggests that the tissues and time points we selected for analysis were appropriate, and the differentially expressed genes identified through RNA‐seq are reliable. The differentially expressed genes are enriched in several pathways related to the formation and development of the mammary placodes, including the WNT and Notch signaling pathways (Boras‐Granic et al. 2006; Buono et al. 2006). This suggests that the differentially expressed genes may exert their effects through these classic signaling pathways crucial for mammary placodes formation, leading to differences in teat number phenotypes between breeds.

In fact, cross‐breed comparisons can introduce many false positives. Erhualian and Bamaxiang pigs differ not only in teat number phenotype but also in litter size, body size, and other traits (China National Committee on Animal Genetic Resources 2011). Therefore, multi‐omics approaches are needed to further identify key genes. In this study, by intersecting genes annotated from differential chromatin accessibility regions identified by ATAC‐seq with differentially expressed genes identified from RNA‐seq, we identified three protein‐coding genes, *ENSSSCG00000031037*, *ENSSSCG00000032042*, and *ENSSSCG00000039180*, which showed significant signals in both analyses and are adjacent to each other. Currently, there is a lack of research on these three genes. To further determine their function, we performed pheWAS analysis using the PigBioBank database and found that these genes are significantly associated with teat number traits in Western commercial pigs, including Duroc, Large White, and Landrace pigs. The teat number GWAS statistics in the Duroc/Landrace/Yorkshire populations also showed genome‐wide significant loci in the regions where these genes are located. The GWAS results based on Chinese‐Western hybrid pig breeds, with Chinese indigenous pig breeds such as Erhualian pigs as the maternal lineage, revealed a significant association between this region and total teat number in these hybrids. This suggests that this region may exert an influence on the total teat number phenotype across various Chinese‐Western pig breeds, including Erhualian pigs.

In the aforementioned region, due to strong selection pressure, the LD in Erhualian pigs increased from the genome‐wide average of *r* ^2^_0.3_ = 9.5 kb to *r* ^2^_0.3_ > 500 kb. Consequently, this region represents a highly linked genomic segment in the Erhualian pig population. Additionally, the three genes are located in close genomic proximity: *ENSSSCG00000031037* is positioned at SSC14: 48800256 bp‐48,800,764 bp, *ENSSSCG00000032042* at SSC14: 48819370 bp‐48,820,324 bp, and *ENSSSCG00000039180* at SSC14: 48824977 bp‐48,825,547 bp, making them adjacent to one another. Furthermore, RNA‐seq data revealed that these three genes exhibit highly similar expression patterns (Figure 6A,C,E). Genomically adjacent genes are often regulated by the same cis‐regulatory elements, forming coexpression modules that likely share similar biological functions, potentially leading to functional redundancy. Additionally, RNA‐seq data indicated that *ENSSSCG00000031037* exhibits the highest expression among the three genes (Figure 6A). Since FISH signal intensity correlates directly with target RNA abundance, selecting this gene ensures a clear probe signal, minimizing the risk of false negatives associated with low‐expression genes. Finally, FISH experiments located the mRNA expression site of *ENSSSCG00000031037* and found that it is specifically expressed near the ectoderm in mammary placodes. These multiple results suggest that the three key genes identified through multi‐omics in this study may be involved in the formation and development of pig mammary placodes, thereby affecting the teat number phenotype.

In addition, we further explored the potential genomic reasons behind the variation in teat number between breeds. By detecting selection signals in the vicinity of the SSC14 48.80 Mb region where these three genes are located, we found significant selection signals in Erhualian pigs. In fact, Erhualian pigs are an indigenous breed developed through long‐term selective breeding by residents of the Taihu Lake region, characterized by high litter size and numerous teats (China National Committee on Animal Genetic Resources 2011; Li et al. 2017). This selective pressure may be concentrated in the genomic regions around the chromatin accessible regions in embryonic day 26 mammary placodes tissue, thereby altering the expression of genes near these accessible regions and leading to the high teat number phenotype in Erhualian pigs.

This study focused on Erhualian pigs, a representative Chinese breed with a high number of teats, and Bamaxiang pigs, a breed with a low number of teats, to preliminarily elucidate the molecular regulatory mechanisms underlying teat number traits. Given the critical impact of teat number on piglet health and survival rate, we employed ATAC‐seq and RNA‐seq to analyze mammary placodes at embryonic day 26 and identified three candidate genes significantly associated with teat number: *ENSSSCG00000031037, ENSSSCG00000032042*, and *ENSSSCG00000039180*. Notably, we confirmed the specific expression of *ENSSSCG00000031037* in mammary placode epithelial cells. These findings not only contribute to the identification of causal genes within QTL regions but also provide a theoretical foundation for the future application of marker‐assisted selection or gene‐editing technologies in pig breeding. Such advancements would facilitate the rapid screening of superior genetic backgrounds, ultimately enhancing sow lactation capacity, improving piglet survival rates, and promoting piglet health. The next step is to further elucidate the genetic mechanisms by which these candidate genes exert their functions and to identify key causal variants, thereby establishing a foundation for practical breeding applications in pig farms.

## 5 Conclusions

In this study, we conducted ATAC‐seq on embryonic day 26 mammary placodes from Erhualian pigs with extremely high teat numbers and Bamaxiang pigs with extremely low teat numbers. This is the first time an accessibility map of pig mammary placodes tissue has been constructed, revealing a broad distribution of chromatin accessibility regions across both autosomes and the X chromosome. The minimal accessibility differences between Erhualian and Bamaxiang pigs suggest a degree of conservation in chromatin accessibility within the mammary placodes across different pig breeds. However, the large number of differentially expressed genes between these two breeds indicates distinct gene expression patterns at the transcriptional level. Through the intersection analysis of ATAC‐seq, RNA‐seq data, pheWAS, and GWAS statistics, along with gene expression validation using FISH experiments and selection signal analyses, we identified three protein‐coding genes, *ENSSSCG00000031037*, *ENSSSCG00000032042*, and *ENSSSCG00000039180*, as key candidate genes responsible for the variation in teat number between Erhualian and Bamaxiang pigs.

## Ethics Statement

All experimental animal procedures in this research adhered to the Guidelines for the Management of Experimental Animals as mandated by China. This study was ethically approved by the committee at Nanjing Agricultural University, under approval ID SYXK‐2021‐0086.

## Conflicts of Interest

The authors declare no conflicts of interest.

## Supporting information


Data S1.

## Data Availability

The RNA‐seq datasets used and analyzed during this study are available in the NCBI SRA: PRJNA1145140. The ATAC‐seq datasets used and analyzed during this study are available in the NCBI SRA: PRJNA1146936.
