# Supplementary material for: Integration ATAC‐Seq and RNA‐Seq Analysis of Mammary Placodes in Erhualian and Bamaxiang Pigs Identified Candidate Genes Influencing Pig Teat Number Variation
Source: Evol Appl. 2025 Jun 28;18(7):e70129. doi: 10.1111/eva.70129 (PMC12205214; doi:10.1111/eva.70129)
Supplement: Supplementary file 1 — Data S1. [file EVA-18-e70129-s001.docx]

Overview List of Supplementary Figures and Tables

**Figure S1.** Comparison of actual and expected values of NRF, PCB1, and PCB2 metrics in ATAC-seq of mammary placodes from 3 Erhualian pigs and 3 Bamaxiang pigs.

**Figure S2.** Distribution of insert sizes in ATAC-seq of mammary placodes from 3 Erhualian Pigs and 3 Bamaxiang Pigs.

**Figure S3.** Enrichment results near transcription start sites (TSS) in mammary placodes of 3 Erhualian pigs and 3 Bamaxiang pigs.

**Figure S4.** Clustering heatmap of gene expression levels based on RNA-seq data from embryonic day 26 mammary placodes.

**Figure S5.** Linkage disequilibrium (LD) extent plot for the 48.80 Mb region on SSC14 in Erhualian and Bamaxiang pig populations.

**Figure S6.** Manhattan plot of Fst analysis between Erhualian and Bamaxiang pigs.

**Table S1.** Statistics of differential chromatin accessibility regions between Erhualian and Bamaxiang pigs.

**Table S2.** Statistics of gene annotation results within 3 kb of differential chromatin accessibility regions between Erhualian and Bamaxiang pigs.

**Table S3.** KEGG enrichment analysis of differentially expressed genes from RNA-seq data.

**Table S4.** KEGG and GO enrichment analysis of differentially expressed genes that significantly interact with *OLIG2* and *NEUROD2.*


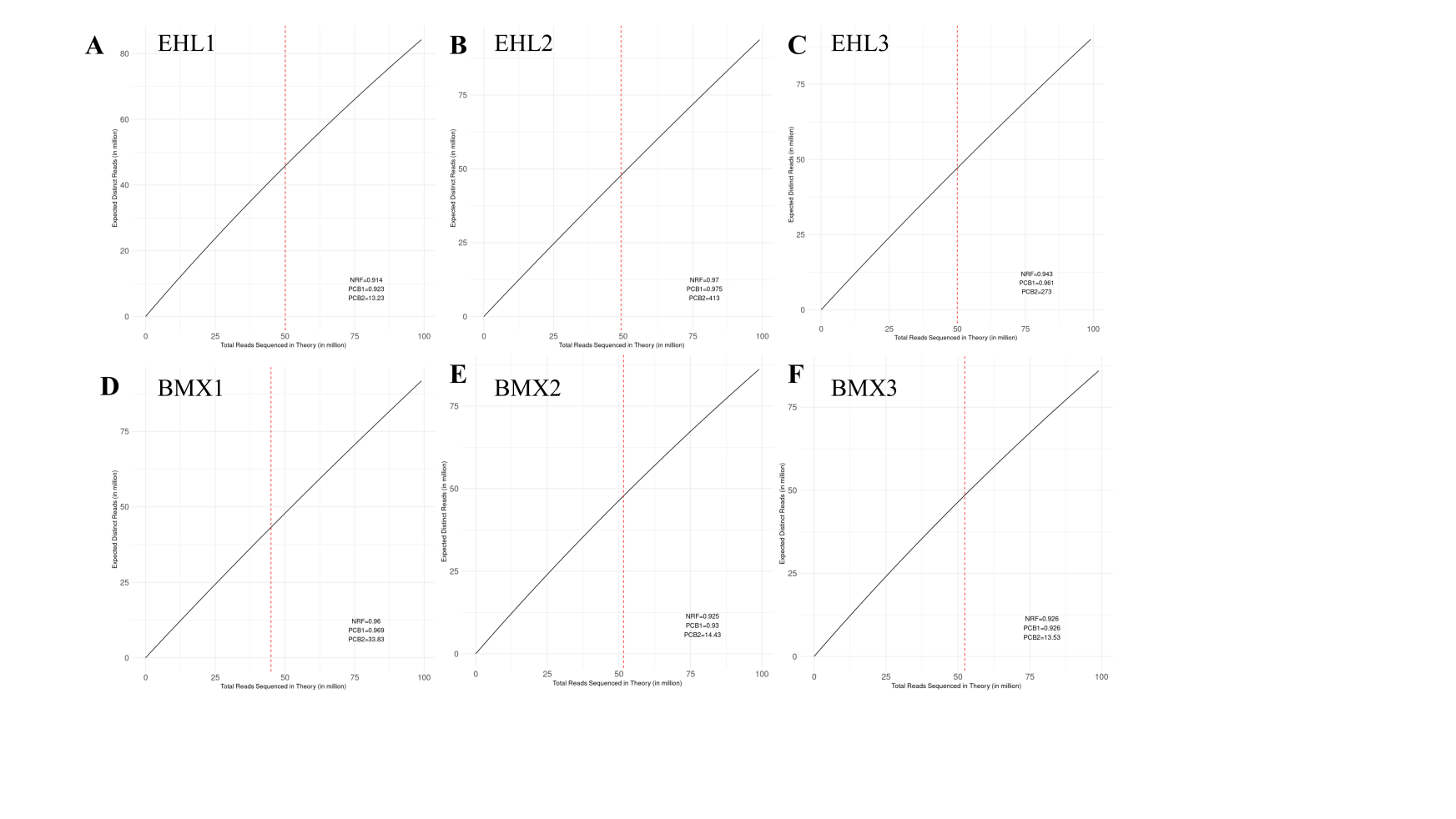


**Figure S1.** Comparison of actual and expected values of NRF, PCB1, and PCB2 metrics in ATAC-seq of mammary placodes from 3 Erhualian pigs and 3 Bamaxiang pigs. (A)-(C) Correspond to Erhualian pigs 1-3. (D)-(F) Correspond to Bamaxiang pigs 1-3. The x-axis represents Total Reads Sequenced in Theory, and the y-axis represents expected distinct reads. NRF, PCB1, and PCB2 stand for Non-Redundant Fraction, Phantom Peak Coefficient 1, and Phantom Peak Coefficient 2, respectively.


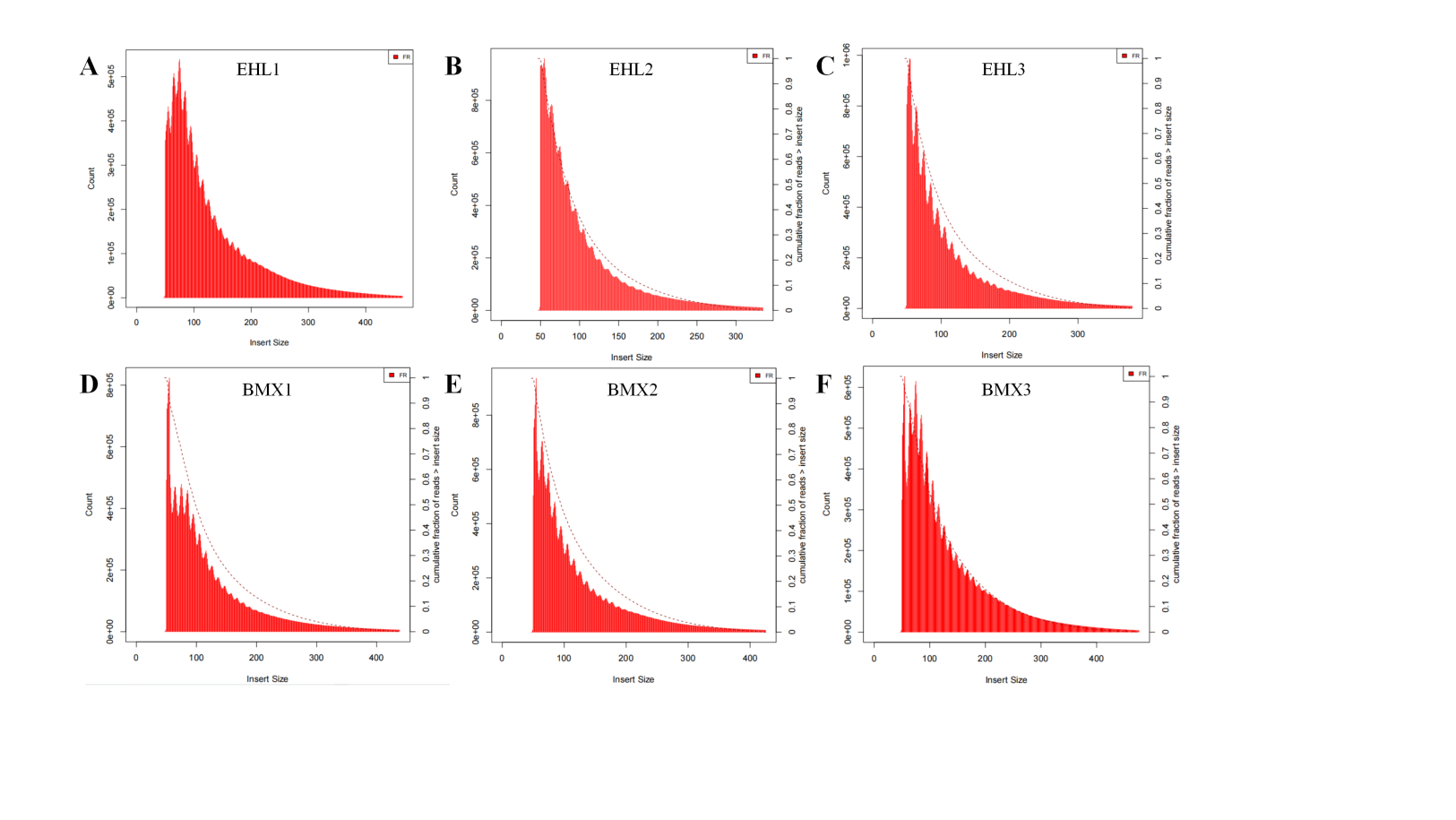


**Figure S2.** Distribution of insert sizes in ATAC-seq of mammary placodes from 3 Erhualian Pigs and 3 Bamaxiang Pigs. (A)-(C) Correspond to Erhualian pigs 1-3. (D)-(F) Correspond to Bamaxiang pigs 1-3. The x-axis represents the insert size of reads, and the y-axis represents the count of corresponding reads.


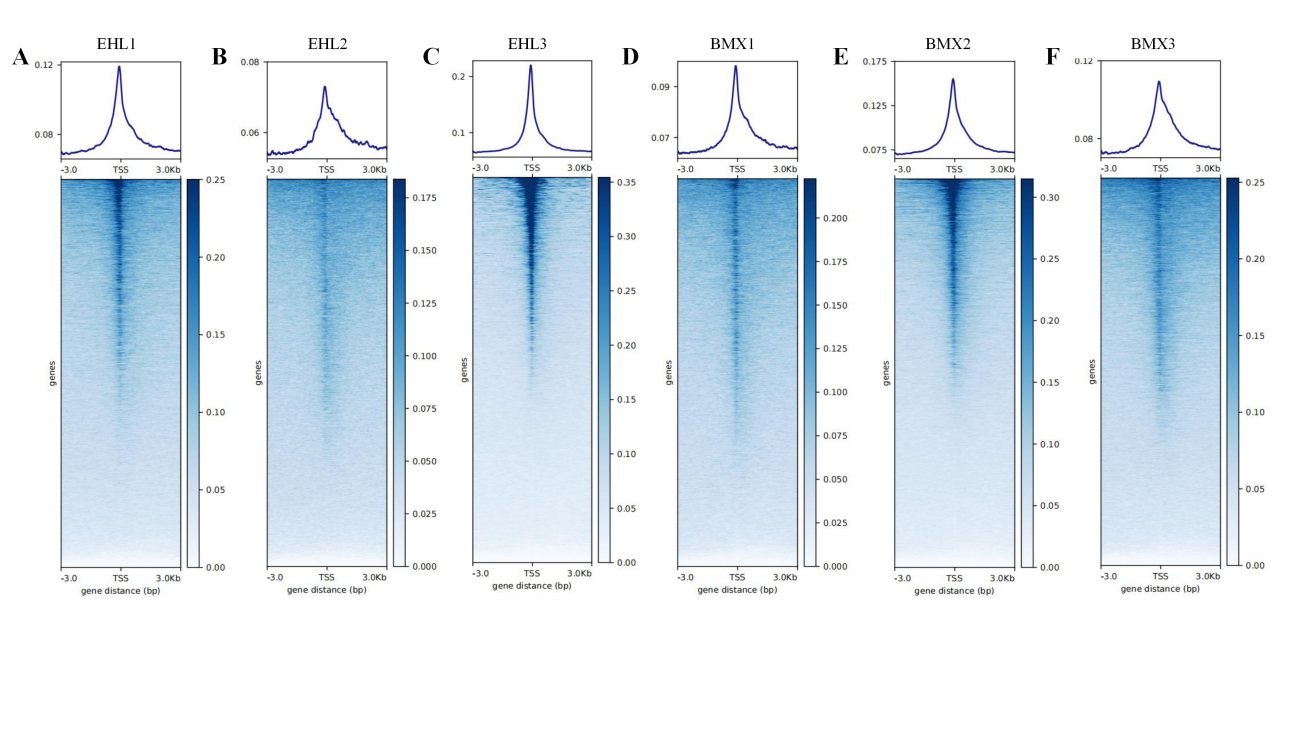


**Figure S3.** Enrichment results near transcription start sites (TSS) in mammary placodes of 3 Erhualian pigs and 3 Bamaxiang pigs. (A)-(C) Correspond to Erhualian pigs 1-3. (D)-(F) Correspond to Bamaxiang pigs 1-3. Darker blue indicates a higher enrichment rate.


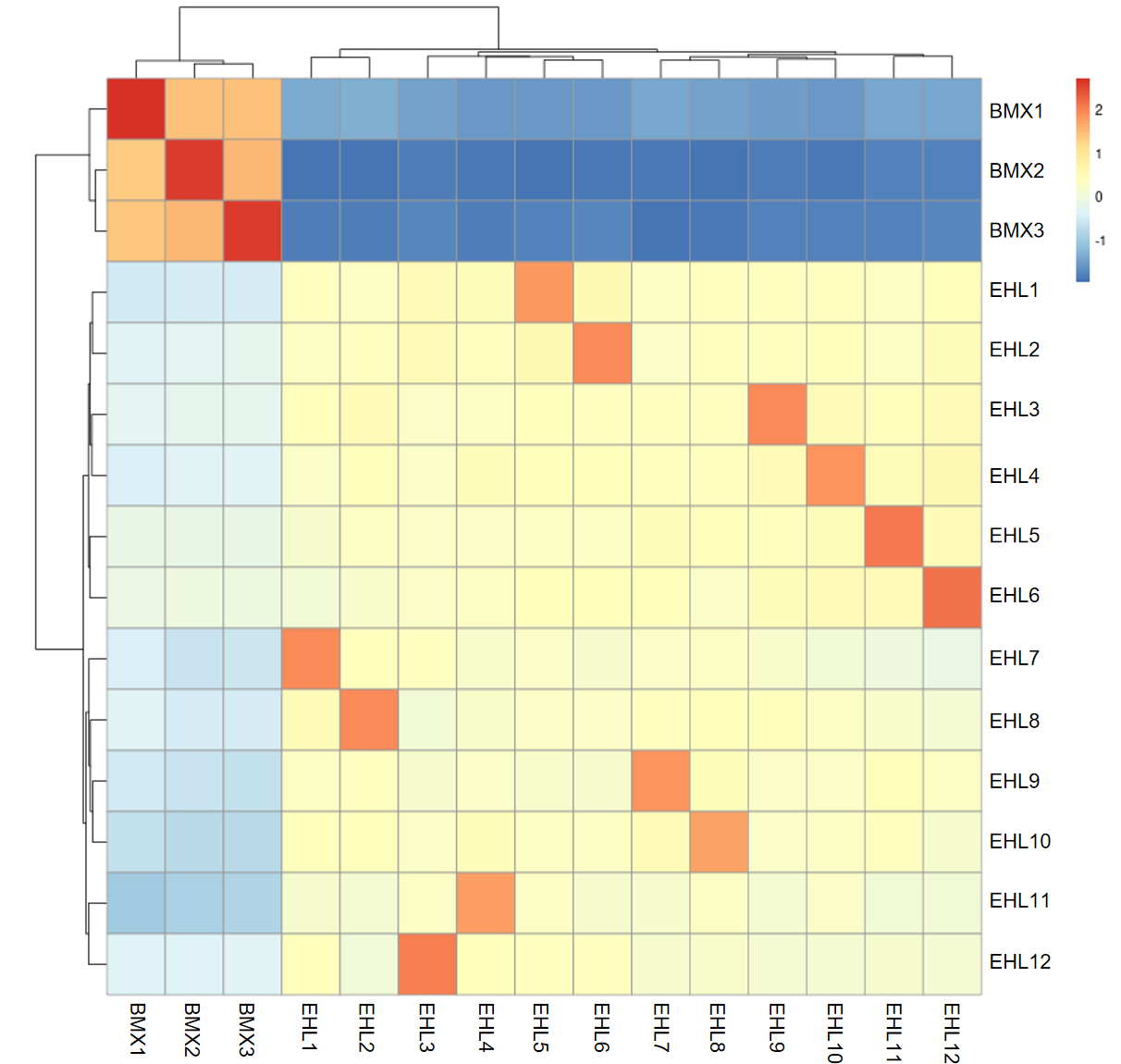


**Figure S4.** Clustering heatmap of gene expression levels based on RNA-seq data from embryonic day 26 mammary placodes. EHL represents Erhualian pigs, and BMX represents Bamaxiang pigs. The color gradient from blue to red indicates an increasing correlation coefficient among samples.

**Figure S5.** Linkage disequilibrium (LD) extent plot for the 48.80 Mb region on SSC14 in Erhualian and Bamaxiang pig populations. Inter-SNP distances are plotted along the x-axis, while LD extents (predicted by r²) are plotted along the y-axis. The red solid line indicates the threshold of 0.3.


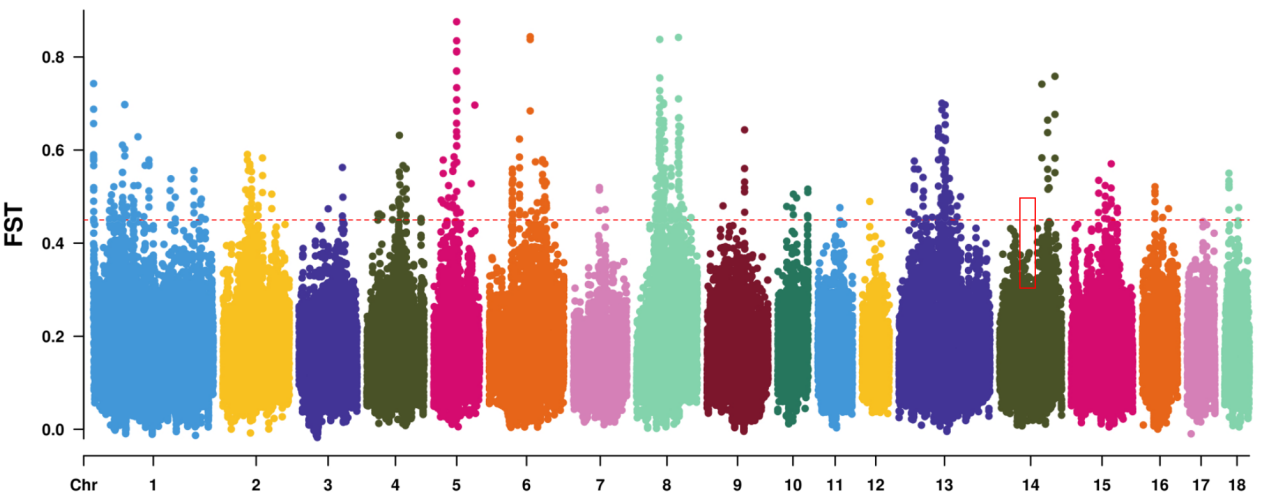


**Figure S6.** Manhattan plot of Fst analysis between Erhualian and Bamaxiang pigs. The red dashed line marks the significance threshold (top 5%, Fst value = 0.45), while the red rectangular box highlights the 48.80 Mb region on SSC14.

**Table S1.** Statistics of differential chromatin accessibility regions between Erhualian and Bamaxiang pigs

| seqnames | start | end | Conc^1^ | Conc_A^2^ | Conc_B^3^ | Fold | FDR |
| --- | --- | --- | --- | --- | --- | --- | --- |
| 5 | 40182453 | 40182953 | 7.494018028 | 8.384901061 | 4.714984384 | 3.470805456 | 1.73E-08 |
| 5 | 40020985 | 40021485 | 4.950435376 | 5.848328355 | 2.079070577 | 3.522275085 | 3.15E-06 |
| 14 | 6355947 | 6356447 | 4.539362364 | 5.539362364 | 0 | 6.95110988 | 3.15E-06 |
| 5 | 40188592 | 40189092 | 8.310947858 | 9.158758956 | 5.990690965 | 2.979020952 | 3.15E-06 |
| 5 | 40184373 | 40184873 | 7.416510055 | 8.28384638 | 4.907765413 | 3.158353251 | 3.29E-06 |
| 5 | 40190133 | 40190633 | 8.690720283 | 9.533969647 | 6.410831362 | 2.925025455 | 1.30E-05 |
| 16 | 47477715 | 47478215 | 6.079676769 | 5.102864693 | 6.656826395 | -1.516767763 | 0.000234392 |
| 13 | 58338 | 58838 | 4.571100158 | 5.559054414 | 0 | 5.713721703 | 0.000261799 |
| 5 | 40092960 | 40093460 | 5.860103492 | 6.512957213 | 4.634858397 | 1.795391648 | 0.000560559 |
| 14 | 48824532 | 48825032 | 5.727073267 | 6.361478161 | 4.567686185 | 1.686110123 | 0.00215512 |
| X | 47933992 | 47934492 | 4.886704392 | 3.502469696 | 5.579941393 | -1.957393431 | 0.002550316 |
| 2 | 71255595 | 71256580 | 6.061237521 | 6.687359292 | 4.930207294 | 1.619863501 | 0.002550316 |
| 14 | 31804643 | 31805143 | 4.495347772 | 5.345784991 | 2.1512691 | 2.651696262 | 0.003220239 |
| 16 | 47476523 | 47477023 | 5.567115162 | 4.521656909 | 6.16691759 | -1.544307741 | 0.003220239 |
| 12 | 17946705 | 17947205 | 5.421221289 | 4.408783147 | 6.010306121 | -1.53757131 | 0.003285477 |
| 14 | 6353241 | 6353741 | 4.463568429 | 5.279395779 | 2.402825972 | 2.434687992 | 0.003606068 |
| 8 | 130944303 | 130944803 | 5.239527181 | 4.084357956 | 5.872713297 | -1.554454355 | 0.006223599 |
| 5 | 40019914 | 40020414 | 3.824402765 | 4.698728541 | 1.241016092 | 2.435398279 | 0.009313224 |
| 14 | 48798790 | 48799290 | 4.901009696 | 5.578716294 | 3.580541905 | 1.541527045 | 0.010578569 |
| 14 | 6016568 | 6017068 | 4.70062867 | 5.458593358 | 3.005825585 | 1.573307915 | 0.018663558 |
| 8 | 130949950 | 130950450 | 5.349066587 | 4.481742507 | 5.88694643 | -0.963118173 | 0.0199425 |
| 14 | 87396173 | 87396673 | 4.557993402 | 3.190815111 | 5.247159521 | -1.360894581 | 0.020094022 |
| 14 | 48825120 | 48825620 | 5.092667003 | 5.751072163 | 3.846829779 | 1.209264903 | 0.020647024 |
| 12 | 58564616 | 58565116 | 5.558356823 | 6.135328129 | 4.582068209 | 0.914780127 | 0.025195937 |
| 2 | 71257528 | 71258028 | 5.257089269 | 5.88512468 | 4.119571504 | 0.963696848 | 0.02874615 |
| 14 | 48818704 | 48819204 | 5.867478663 | 6.422803152 | 4.95290573 | 0.77194114 | 0.029685639 |
| 5 | 40094582 | 40095082 | 5.015467689 | 5.712350418 | 3.61574327 | 1.027714134 | 0.034141027 |
| 7 | 23996902 | 23997402 | 5.896508302 | 5.166652599 | 6.378877274 | -0.541044439 | 0.034141027 |
| X | 47935852 | 47936352 | 5.030641342 | 4.045173599 | 5.610727045 | -0.739440218 | 0.038654694 |
| 13 | 199677064 | 199677564 | 4.892470878 | 5.80400055 | 1.821032863 | 1.045357263 | 0.047859937 |

^1^ representing the normalized signal intensity or concentration of reads at a given peak, indicating the accessibility of that region of the genome. ^2^ represents the accessibility level in Erhualian pigs. ^3^ represents the accessibility level in Bamaxiang pigs.

**Table S2.** Statistics of gene annotation results within 3 kb of differential chromatin accessibility regions between Erhualian and Bamaxiang pigs

| Gene stable ID | CHR | Gene start (bp) | Gene end (bp) | Gene type | Gene name |
| --- | --- | --- | --- | --- | --- |
| ENSSSCG00000017577 | 12 | 17864029 | 17963155 | protein_coding |  |
| ENSSSCG00000062975 | 12 | 58566073 | 58566170 | miRNA | ssc-mir-9828-1 |
| ENSSSCG00000051235 | 14 | 6351545 | 6355536 | lncRNA |  |
| ENSSSCG00000031037 | 14 | 48800256 | 48800764 | protein_coding |  |
| ENSSSCG00000032042 | 14 | 48819370 | 48820324 | protein_coding |  |
| ENSSSCG00000039180 | 14 | 48824977 | 48825547 | protein_coding |  |
| ENSSSCG00000016963 | 16 | 47436087 | 47477925 | protein_coding | *CCDC125* |
| ENSSSCG00000037053 | 16 | 47480557 | 47500018 | protein_coding | *AK6* |
| ENSSSCG00000009215 | 8 | 130889684 | 130945393 | protein_coding | *ABCG2* |

**Table S3.** KEGG enrichment analysis of differentially expressed genes from RNA-seq data

| KEGG Term | Input number | Background number | P-Value | Corrected P-Value | Input |
| --- | --- | --- | --- | --- | --- |
| PI3K-Akt signaling pathway | 62 | 354 | 6.92E-12 | 3.14E-10 | PCK2\|NGF\|PCK1\|CHAD\|ERBB3\|ITGA2\|ITGA4\|EPHA2\|TEK\|FGFR4\|NOS3\|NGFR\|LAMB3\|EGF\|FLT1\|COL1A1\|IL7\|PRLR\|LAMA3\|ITGB6\|GNG10\|GNG11\|PIK3CG\|MAGI2\|MYC\|KDR\|PDGFA\|ITGB3\|PDGFC\|ITGB4\|ERBB4\|TNR\|ITGB8\|NR4A1\|PPP2R2C\|PPP2R2B\|COL4A4\|COL4A3\|CSF3R\|ATF4\|FGF6\|KITLG\|FGFR3\|TCL1B\|TCL1A\|SGK3\|SGK2\|SGK1\|FLT4\|IFNA1\|FGF23\|IL6\|CREB3L3\|COL1A2\|TLR4\|EIF4E1B\|COL6A1\|FASLG\|VWF\|PPP2R5A\|GNG8\|MCL1 |
| Wnt signaling pathway | 28 | 160 | 3.75E-06 | 1.89E-05 | WIF1\|CER1\|FZD10\|FZD2\|FZD5\|FZD7\|WNT10A\|WNT10B\|JUN\|SOX17\|FOSL1\|MYC\|WNT2B\|PLCB1\|PLCB2\|RAC2\|CAMK2B\|PRKCG\|LEF1\|NOTUM\|SFRP5\|WNT3\|WNT6\|WNT4\|FRAT1\|FRAT2\|WNT7A\|WNT7B |
| TGF-beta signaling pathway | 19 | 94 | 2.35E-05 | 9.56E-05 | TGFB1\|GREM1\|HAMP\|BMP6\|NBL1\|ID4\|AMHR2\|ID1\|TGFBR2\|BMPR1B\|TNF\|FST\|NOG\|PITX2\|AMH\|MYC\|SMAD6\|TGIF1\|CHRD |
| ECM-receptor interaction | 18 | 86 | 2.56E-05 | 0.000101269 | COL1A1\|ITGB3\|LAMA3\|ITGB4\|CHAD\|ITGB6\|SDC1\|ITGA2\|TNR\|ITGA4\|NPNT\|COL4A4\|COL4A3\|ITGB8\|COL6A1\|COL1A2\|LAMB3\|VWF |
| Estrogen signaling pathway | 22 | 138 | 0.000132487 | 0.000396211 | TFF1\|JUN\|PLCB1\|PLCB2\|NOS3\|FOS\|CTSD\|KRT26\|KRT24\|ITPR2\|ITPR1\|ADCY4\|ESR1\|KCNJ9\|ADCY8\|CALML5\|CALML4\|KRT17\|CREB3L3\|KRT15\|ATF4\|MMP2 |
| Notch signaling pathway | 9 | 48 | 0.004978474 | 0.009338321 | DTX3L\|DTX4\|RBPJL\|MFNG\|NOTCH4\|DLL4\|PTCRA\|RFNG\|NUMBL |

**Table S4.** KEGG and GO enrichment analysis of differentially expressed genes that significantly interact with *OLIG2* and *NEUROD2*

| KEGG/GO Term | Database | Input number | Background number | P-Value | Corrected P-Value | Input |
| --- | --- | --- | --- | --- | --- | --- |
| Estrogen signaling pathway | KEGG PATHWAY | 4 | 138 | 2.08E-04 | 2.41E-03 | ITPR2\|PLCB1\|KCNJ9\|PLCB2 |
| Parathyroid hormone synthesis, secretion and action | KEGG PATHWAY | 3 | 106 | 1.46E-03 | 1.06E-02 | ITPR2\|PLCB1\|PLCB2 |
| PI3K-Akt signaling pathway | KEGG PATHWAY | 4 | 354 | 6.21E-03 | 3.24E-02 | ERBB3\|FGFR4\|LAMA3\|PDGFC |
| EGFR tyrosine kinase inhibitor resistance | KEGG PATHWAY | 2 | 79 | 1.19E-02 | 4.79E-02 | ERBB3\|PDGFC |
| embryo development | Gene Ontology | 2 | 17 | 6.83E-04 | 5.71E-03 | DLX1\|DLX2 |
| Wnt signaling pathway, calcium modulating pathway | Gene Ontology | 2 | 37 | 2.88E-03 | 1.80E-02 | PLCB1\|PLCB2 |
| negative regulation of Notch signaling pathway | Gene Ontology | 2 | 38 | 3.03E-03 | 1.87E-02 | DLX1\|DLX2 |
| estrogen receptor binding | Gene Ontology | 2 | 42 | 3.66E-03 | 2.17E-02 | ISL1\|PPARGC1B |
| positive regulation of epithelial cell proliferation | Gene Ontology | 2 | 59 | 6.92E-03 | 3.53E-02 | IHH\|NOG |
| response to estrogen | Gene Ontology | 2 | 61 | 7.37E-03 | 3.67E-02 | GATA6\|ABCC2 |
| epithelial cell maturation | Gene Ontology | 1 | 5 | 1.21E-02 | 4.79E-02 | TFCP2L1 |
| ErbB-3 class receptor binding | Gene Ontology | 1 | 5 | 1.21E-02 | 4.79E-02 | ERBB3 |
| mesenchymal cell differentiation | Gene Ontology | 1 | 7 | 1.62E-02 | 4.83E-02 | ISL1 |
